# Gaussian Graphical Models Reveal Inter-Modal and Inter-Regional Conditional Dependencies of Brain Alterations in Alzheimer's Disease

**DOI:** 10.3389/fnagi.2020.00099

**Published:** 2020-04-21

**Authors:** Martin Dyrba, Reza Mohammadi, Michel J. Grothe, Thomas Kirste, Stefan J. Teipel

**Affiliations:** ^1^German Center for Neurodegenerative Diseases (DZNE), Rostock, Germany; ^2^Department of Operation Management, Amsterdam Business School, University of Amsterdam, Amsterdam, Netherlands; ^3^Mobile Multimedia Information Systems Group (MMIS), University of Rostock, Rostock, Germany; ^4^Clinic for Psychosomatics and Psychotherapeutic Medicine, Rostock University Medical Center, Rostock, Germany

**Keywords:** Alzheimer's disease, mild cognitive impairment, conditional dependency networks, Gaussian graphical models, graph-theoretical analysis, small-world network

## Abstract

Alzheimer's disease (AD) is characterized by a sequence of pathological changes, which are commonly assessed *in vivo* using various brain imaging modalities such as magnetic resonance imaging (MRI) and positron emission tomography (PET). Currently, the most approaches to analyze statistical associations between regions and imaging modalities rely on Pearson correlation or linear regression models. However, these models are prone to spurious correlations arising from uninformative shared variance and multicollinearity. Notably, there are no appropriate multivariate statistical models available that can easily integrate dozens of multicollinear variables derived from such data, being able to utilize the additional information provided from the combination of data sources. Gaussian graphical models (GGMs) can estimate the conditional dependency from given data, which is conceptually expected to closely reflect the underlying causal relationships between various variables. Hence, we applied GGMs to assess multimodal regional brain alterations in AD. We obtained data from *N* = 972 subjects from the Alzheimer's Disease Neuroimaging Initiative. The mean amyloid load (AV45-PET), glucose metabolism (FDG-PET), and gray matter volume (MRI) were calculated for each of the 108 cortical and subcortical brain regions. GGMs were estimated using a Bayesian framework for the combined multimodal data and the resulted conditional dependency networks were compared to classical covariance networks based on Pearson correlation. Additionally, graph-theoretical network statistics were calculated to determine network alterations associated with disease status. The resulting conditional dependency matrices were much sparser (≈10% density) than Pearson correlation matrices (≈50% density). Within imaging modalities, conditional dependency networks yielded clusters connecting anatomically adjacent regions. For the associations between different modalities, only few region-specific connections were detected. Network measures such as small-world coefficient were significantly altered across diagnostic groups, with a biphasic u-shape trajectory, i.e., increased small-world coefficient in early mild cognitive impairment (MCI), similar values in late MCI, and decreased values in AD dementia patients compared to cognitively normal controls. In conclusion, GGMs removed commonly shared variance among multimodal measures of regional brain alterations in MCI and AD, and yielded sparser matrices compared to correlation networks based on the Pearson coefficient. Therefore, GGMs may be used as alternative to thresholding-approaches typically applied to correlation networks to obtain the most informative relations between variables.

## 1. Introduction

Alzheimer's disease (AD) is characterized by a range of pathological brain alterations that can be assessed *in vivo* using various neuroimaging methods, including MRI and PET. Several studies suggest that information obtained from combining different imaging modalities could provide reliable markers of cerebral reserve capacity and might be used to predict and monitor the evolution of AD and its relative impact on cognitive domains in pre-clinical, prodromal, and dementia stages of AD [see e.g., reviews (Teipel S. et al., 2015; Teipel et al., 2016)]. However, there is still an unmet need for appropriate analysis methods for assessing statistical associations between individual brain regions and between different pathology markers derived from multiple neuroimaging modalities.

Up to date, multimodal studies employ one of the following approaches:

- Correlation of pathology maps on a voxel level (La Joie et al., 2012; Altmann et al., 2015; Grothe and Teipel, 2016);
- linear regression analysis with a-priori specified regions-of-interest (Buckner et al., 2005; Seeley et al., 2009; Villain et al., 2010; Kljajevic et al., 2014; Chang et al., 2015; Grothe et al., 2016; Teipel and Grothe, 2016);
- stratification of subjects into distinct groups (e.g., amyloid positive/negative) to compare differences in other imaging modalities (Buckner et al., 2005; Kljajevic et al., 2014; Grothe et al., 2016);
- comparison of graph-theoretical measures and statistics between modalities (Stam et al., 2006; Buckner et al., 2009; Zhou et al., 2012; Sepulcre et al., 2013, 2017); and
- estimation of generative models for comparing spreading mechanisms of amyloid-β deposition and its contribution to neurodegeneration (Dyrba et al., 2017; Iturria-Medina et al., 2017; Torok et al., 2018).

Commonly employed statistical models, such as linear regression analysis, provide limited ability to assess the interactions between dozens of variables in the same model, as they cannot derive reliable estimates regarding the individual contribution of highly collinear predictors and suffer from variance inflation (Dormann et al., 2013). Calculation of covariance/connectivity matrices based on the Pearson correlation between each pair of variables has led to practical problems in deriving meaningful results, i.e., these matrices are commonly thresholded to an a-priori defined density and binarized (Buckner et al., 2009; Zhou et al., 2012; Sepulcre et al., 2013). More recently, summary statistics based on graph-theory have been proposed (Watts and Strogatz, 1998; Stam et al., 2006) and are currently widely applied (Buckner et al., 2009; Zhou et al., 2012; Sepulcre et al., 2013, 2017). However, this approach has been criticized, as for instance, group differences in small-worldness of the brain network might be sensitive to the specific density threshold (Hlinka et al., 2017; Mårtensson et al., 2018).

**Figure 1:**
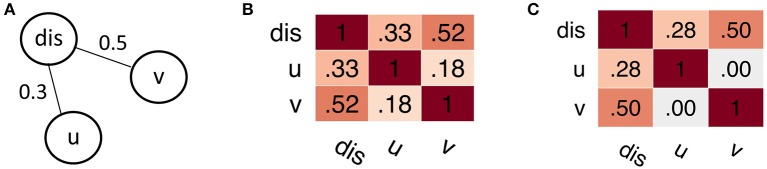
Simple example for spurious correlations. **(A)** True dependency graph. The node *u* is statistically independent from *v* given the node *dis*, formally *p*(*u, v*|*dis*) = *p*(*u*|*dis*)*p*(*v*|*dis*). **(B)** Pearson correlation matrix, showing a “spurious” correlation between nodes u and v. Notably, when considering only *u* and *v* alone, the independence assumption does not hold; formally *p*(*u, v*) ≠ *p*(*u*)*p*(*v*). **(C)** Partial correlation matrix derived from Gaussian graphical models. Using this model, we can approximately recover the underlying dependency structure, with *u*⊥*v*|*dis* ⇒ *cor*(*u, v*|*dis*) = 0.

We suggest the application of Gaussian graphical models (GGMs), which are able to estimate the *partial* correlation between various multicollinear predictors (Hastie et al., 2013, chapter 7.3). GGMs yield sparse conditional dependency matrices, that are conceptually expected to closer reflect the underlying causal relationships (Koller and Friedman, 2009, chapter 21.7; Bontempi and Flauder, 2015). This makes GGMs an interesting candidate for studying properties of the brain network; an example is illustrated in Figure 1. The partial correlation derived from GGMs is conceptually similar to the partial correlation obtained from a series of linear regression models, which estimate the statistical association of the dependent and independent variables while controlling for the confounding variables. Additionally, GGMs extend this concept by estimating the partial correlation matrix as a set of coupled regression problems, in contrast to separate regression problems modeled by traditional linear regression (Meinshausen and Bühlmann, 2006; Hastie et al., 2013, chapter 7.3). Technically, GGMs are naively realized by matrix inversion of the covariance matrix. In more robust and efficient approaches, regularization techniques (Meinshausen and Bühlmann, 2006; Ravikumar et al., 2011; Ryali et al., 2012; Cai et al., 2013; Wang et al., 2016) or efficient sampling schemes (Mohammadi and Wit, 2015, 2019) are applied.

In this paper, we tested the applicability and clinical utility of GGMs to reveal the conditional dependency structure between regional pathology measures. For this purpose, we assessed inter-regional statistical associations within and between three main imaging markers of Alzheimer's disease using GGMs based on a whole-cortex parcellation of the brain. The assessed imaging markers included amyloid-β deposition (florbetapir/AV45-PET), glucose metabolism (FDG-PET), and gray matter volume (*T*_1_-weighted MRI). Based on our previous results with only six representative brain regions (Dyrba et al., 2017), we hypothesized that regional amyloid deposition has low contribution to gray matter atrophy, whereas hypometabolism was expected to be stronger related to atrophy. Further, we expected a few hub-nodes influencing pathology in other regions. For graph-theoretical measures, we expected a linear trajectory of decreasing clustering coefficient and increasing path length with stronger disease severity, as previously reported in the literature for connectivity analyses based on Pearson correlation (He et al., 2008; Yao et al., 2010; Li et al., 2012; Morbelli et al., 2012; Tijms et al., 2013; Pereira et al., 2016; John et al., 2017; Titov et al., 2017).

## 2. Materials and Methods

### 2.1. Study Participants

Data were obtained from the Alzheimer's Disease Neuroimaging Initiative (ADNI) database (http://adni.loni.usc.edu). The ADNI was launched in 2003 by the National Institute on Aging, the National Institute of Biomedical Imaging and Bioengineering, the Food and Drug Administration, private pharmaceutical companies, and non-profit organizations, with the primary goal of testing whether neuroimaging, neuropsychological, and other biological measurements can be used as reliable *in vivo* markers of Alzheimer's disease pathogenesis. A complete description of ADNI and up-to-date information is available at http://www.adni-info.org. For this study, 529 subjects with amnestic mild cognitive impairment (MCI), 189 patients with Alzheimer's dementia (AD), and 254 cognitively healthy control subjects (CN) were selected from the ADNI-GO, ADNI-2, and ADNI-3 extensions of the ADNI project, based on the availability of concurrent structural MRI, FDG-PET, amyloid-sensitive AV45-PET, and neuropsychological assessments. In ADNI, two MCI subgroups exist, which only differ by the less severe impairment of memory function for *early MCI* (EMCI) compared to *late MCI* (LMCI) subjects. Detailed inclusion criteria for the diagnostic categories can be found at the ADNI website (http://adni.loni.usc.edu/methods, ADNI2 manual page 27). Demographics and neuropsychological profiles of the different diagnostic groups are summarized in Table 1.

**Table 1:** Sample characteristics.

	**CN**	**EMCI**	**LMCI**	**AD**
Sample size (female)	254(130)	309(135)	220(93)	189(80)
Age (SD)	75.4 ± 6.6	71.6 ± 7.5*	74.1 ± 8.1	75.0 ± 8.0
Education (SD)	16.4 ± 2.7	16.0 ± 2.6	16.2 ± 2.8	15.9 ± 2.7
MMSE (SD)	29.1 ± 1.2	28.3 ± 1.6*	27.6 ± 1.9*	22.6 ± 3.2*
Delayed recall (SD)	7.6 ± 4.1	5.7 ± 4.0*	3.2 ± 3.7*	0.8 ± 1.9*

Gender distribution did not differ significantly between groups (P = 0.15, chi-square test).

*Asterisks indicate significant difference between groups (P < 0.05) based on pairwise two-sample t-test with CN as reference group. CN, cognitively healthy elderly controls; EMCI/LMCI, early and late amnestic mild cognitive impairment; AD, Alzheimer's dementia; MMSE, Mini-Mental State Examination; delayed recall, number of remembered words out of a 15-item wordlist of the Rey Auditory Verbal Learning Test*.

### 2.2. Imaging Data and Feature Extraction

ADNI-GO/2 MRI, FDG- and AV45-PET data were downloaded from the ADNI image archive. ADNI-GO/2 MRI data were acquired on multiple 3T MRI scanners using scanner-specific T1-weighted sagittal 3D MP-RAGE/IR-SPGR sequences. To increase signal uniformity across the multicenter scanner platforms, original T1 acquisitions underwent standardized image preprocessing correction steps (http://adni.loni.usc.edu/methods/mri-tool/mri-pre-processing/). FDG- and AV45-PET data were acquired on multiple instruments of varying resolution and following different platform-specific acquisition protocols. Similar to the MRI data, PET data in ADNI were also subject to standardized image preprocessing correction steps, with the aim of increasing data uniformity across the multicenter acquisitions (http://adni.loni.usc.edu/methods/pet-analysis-method/pet-analysis/). Imaging data were processed by using statistical parametric mapping (SPM8, Wellcome Centre for Human Neuroimaging, University College London) and the VBM8 toolbox (Structural Brain Mapping Group, University of Jena) implemented in MATLAB R2013b (Math-Works, Natick, MA) as previously described in Grothe et al. (2016) and Grothe and Teipel (2016). First, MRI T1 scans were segmented into gray matter, white matter, and cerebrospinal fluid partitions using the segmentation routine of the VBM8 toolbox. Then, the resulting gray matter and white matter segments were spatially normalized to an aging/AD-specific reference template (Grothe et al., 2013) using the DARTEL algorithm. Additionally, voxel values of the normalized gray matter segments were modulated for volumetric changes introduced by the high-dimensional normalization, such that the total amount of gray matter volume present before warping was preserved. Each subject's FDG- and AV45-PET scans were rigidly coregistered to the corresponding skull-stripped T1 scan. Then, the PET scans were corrected for partial volume effects using a three-compartment model and the MRI-derived tissue segments (Müller-Gärtner et al., 1992; Gonzalez-Escamilla et al., 2017). Corrected PET scans were spatially normalized (without modulation) by applying the deformation fields of the T1-weighted scans. All original data and normalized scans were visually inspected to ensure a high quality of the data. Subsequently, mean gray matter volumes and mean FDG-/AV45-PET uptake values were calculated for 108 cortical and subcortical regions defined by the Harvard-Oxford atlas (Desikan et al., 2006) after projecting the atlas to the aging/AD-specific reference space and removing voxels with a gray matter probability of <50% in the aging/AD template. Finally, regional gray matter volumes were proportionally scaled by total intracranial volume (TIV), regional FDG-PET values were proportionally scaled to pons uptake, and regional AV45-PET values were proportionally scaled to whole-cerebellum uptake. To be able to directly compare the different modalities, all regional values were normalized using the congitively normal subjects as reference group (La Joie et al., 2012). As described previously (Dyrba et al., 2017), we used the so-called *W*-scores, which are analogous to *Z*-scores but are adjusted for specific covariates; age, gender, and education in the present case. Like *Z*-scores, *W*-scores have a mean value of 0 and a standard deviation of 1 in the control group, and values of +1.65 and −1.65 correspond to the 95th and 5th percentiles, respectively. To calculate the *W*-scores, regression models were estimated for the control group using age, gender, and education as independent variables and the mean value of each region as dependent variable. Then, *W*-scores were computed using *W* = (*x*_*ij*_−*e*_*ij*_)/*s*_*res, j*_; with *x*_*ij*_ being the *i*th subject's raw value for region *j*; *e*_*ij*_ being the value expected for region *j* in the control group for the *i*th subject's age, gender, and education; and *s*_*res, j*_ being the standard deviation of the residuals for region *j* in controls.

### 2.3. Statistical Modeling

Graphical models provide an effective way for describing statistical patterns in multivariate data and for estimating the conditional dependency between the various brain regions and imaging modalities based on GGMs (Lauritzen, 1996; Mohammadi and Wit, 2015). For data following a multivariate normal distribution, undirected GGMs are commonly used. In these graphical models, the graph structure is directly characterized by the precision matrix, i.e., the inverse of the covariance matrix: non-zero entries in the precision matrix show the edges in the conditional dependency graph. Notably, simple inversion of the covariance matrix usually does not work in real world data sets, as already slight noise in the empirical data causes the precision matrix to contain almost no zero entries. To overcome this problem, regularization techniques or efficient sampling algorithms have been proposed that reduce the effect of noise by additionally employing a sparsity assumption and, thus, only detect the most probable conditional dependencies. For our analyses, we employed a computationally efficient Bayesian framework implemented in the R package BDgraph. More specifically, this framework implements a continuous-time birth-death Markov process for estimating the most probable graph structure and edge weights that correspond to the observed partial correlations (Mohammadi and Wit, 2015, 2019). For this study, BDgraph was substantially extended by multi-threaded parallel processing and marginal pseudo-likelihood approximation to speed up computations.

### 2.4. Experimental Setup

First, we estimated GGMs based on the combined data of EMCI, LMCI, and AD patients to study the conditional dependency between brain regions and modalities. Second, we estimated GGMs for each diagnostic group separately to assess alterations of the graph structures. For the combined model, regional *W*-scores of all MCI and AD patients (*N* = 718) and all three imaging modalities were entered. Initially, we took all 108 cortical and subcortical regions included in the Harvard-Oxford atlas (Desikan et al., 2006) into consideration, corresponding to *P* = 3*108 = 324 variables. The sampling process included 1,000,000 burn-in iterations^1^, starting from a random estimate for the inverse covariance matrix and converging to estimates with higher posterior probability giving the training data. The burn-in iterations were then discarded, and subsequently 150,000 sampling iterations followed to obtain the estimates for the inverse covariance matrix. Because results were showing a strong left–right hemisphere symmetry, we repeated model estimation including only the 54 regions in the left hemisphere (*P* = 3*54 = 162 variables) to increase model stability. From the final model we set a probability threshold of *P*_*avg*_ > 0.5 for selecting the edges, with the notion that a specific edge was considered to be present if it existed in at least half of all model iterations (Madigan et al., 1996). For the second analysis of group differences, we estimated individual GGMs for each group based on the multimodal data of the left hemisphere. Sampling was again performed with 1,000,000 burn-in iterations followed by 150,000 sampling iterations.

For comparison, these analyses were also repeated (i) using data of the right hemisphere to validate the results and (ii) using the traditional approach of constructing correlation networks based on the Pearson correlation coefficient.

### 2.5. Graph-Theoretical Analyses

To assess group differences of the estimated graph structure we calculated the three graph-theoretical measures that are most commonly reported in the literature; clustering coefficient, characteristic path length, and their ratio, the small-world coefficient. The path length quantifies the distance of connections between two nodes along the shortest path. The weighted characteristic path length is the average minimum distance between a node *i*∈*N* and all other nodes, $L_{i}=\underset{j\in N,j\neq i}{\sum }d_{ij}/\left(n-1\right)$, where $d_{ij}=\underset{a_{uv}\in g_{i↔j}}{\sum }\omega_{uv}$ is the shortest weighted path length between *i* and *j*, *g*_*i*↔*j*_ defines the shortest path, and ω_*uv*_ defines the distance between two nodes. Here, the distance matrix was defined as Ω = 1−*abs*(Θ), that is one minus the absolute pair-wise partial correlation as derived from the GGMs or the absolute Pearson coefficient, respectively (Rubinov and Sporns, 2010). The weighted clustering coefficient indicates the inter-connectedness of neighboring nodes *C*_*i*_ = 2*t*_*i*_/(*k*_*i*_(*k*_*i*_−1)), where $t_{i}=0.5\underset{j,h\in N}{\sum }{\left(\omega_{ij}\omega_{ih}\omega_{jh}\right)}^{1/3}$ is the geometric mean of triangles around node *i*, and where $k_{i}=\underset{j\in N}{\sum }a_{ij}$ is the number of nodes connected to node *i* (Onnela et al., 2005; Rubinov and Sporns, 2010). *k*_*i*_ is often referred to as the *degree* of the node *i*, and the link status *a*_*ij*_ = 1 if node *i* is connected to another node *j*, or *a*_*ij*_ = 0 otherwise. The small-world coefficient is defined as the ratio of the clustering coefficient *C* and characteristic path length *L* in comparison to a random network, *S* = (*C*/*C*_*rand*_)/(*L*/*L*_*rand*_), with *S*≫1 in small-world networks (Rubinov and Sporns, 2010). To simplify calculations, we omitted defining a random network to estimate *C*_*rand*_ and *L*_*rand*_, and directly took the ratio *S*_*i*_ = *C*_*i*_/*L*_*i*_ for group comparisons. Notably, we later report the distribution of graph measures for single regions, as the dependency measures were derive from the whole group of subjects. Graph metrics were compared between diagnostic groups using analysis of variance (ANOVA) and Tukey's honest significant difference tests.

## 3. Results

### 3.1. Conditional Dependency of Alzheimer's Pathology

**Figure 2:**
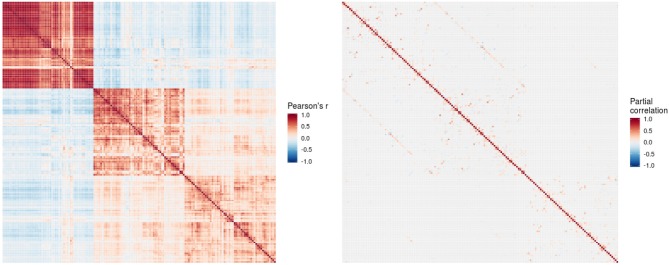
Pearson correlation matrix **(left)** and partial correlation matrix **(right)** for the three imaging modalities (left hemisphere data only) estimated for the combined data of EMCI, LMCI, and AD patients. For better readability, each individual block of the partial correlation matrix is shown in Figures 3–5 and Figures S2–S4. EMCI/LMCI, early and late amnestic mild cognitive impairment; AD, Alzheimer's dementia.

The conditional dependency matrix obtained using the GGM approach for all region of the left hemisphere is given in Figure 2 (right). For the partial correlation between all pairs of brain regions, we obtained 960 significant associations (7% network density) surviving the posterior probability threshold of *P* > 0.5 (see Figure S1 showing the probability of links). For comparison, the Pearson correlation matrix is given in Figure 2 (left). We obtained approximately 6,000 significant Pearson correlations (*P* < 0.05, Bonferroni corrected), corresponding to a network density of 46% of the total number of possible edges.

**Figure 3:**
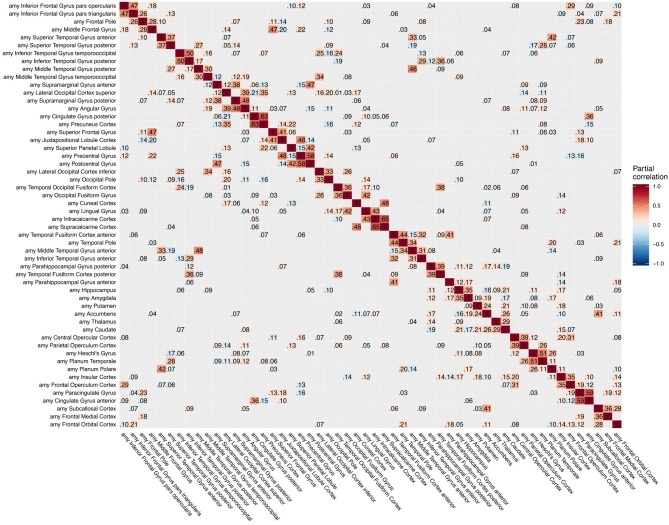
Partial correlation matrix for amyloid-β deposition in the left hemisphere estimated for the combined data of EMCI, LMCI, and AD patients. Averaged over 10 repetitions. Associations of lowest magnitude were not present in all iterations. EMCI/LMCI, early and late amnestic mild cognitive impairment; AD, Alzheimer's dementia; amy, amyloid-β.

**Figure 4:**
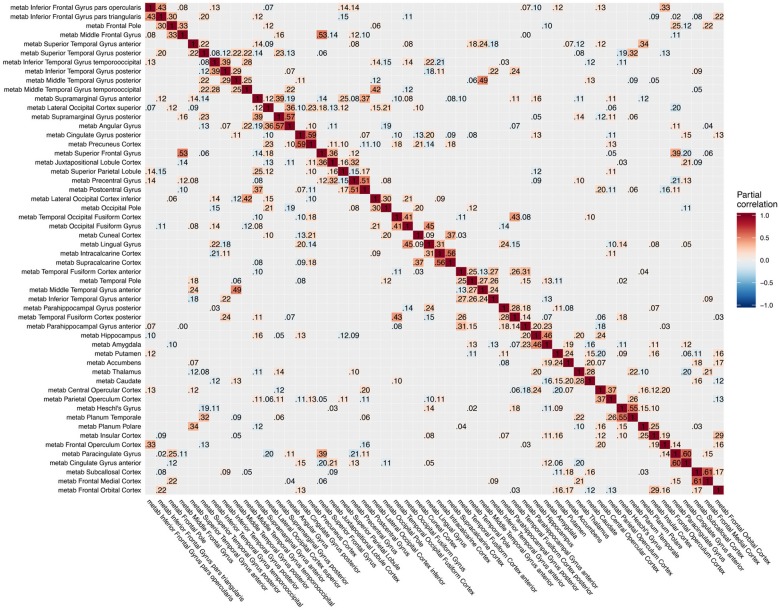
Partial correlation matrix for glucose metabolism in the left hemisphere estimated for the combined data of EMCI, LMCI, and AD patients. Averaged over 10 repetitions. Associations of lowest magnitude were not present in all iterations. EMCI/LMCI, early and late amnestic mild cognitive impairment; AD, Alzheimer's dementia; metab, glucose metabolism.

**Figure 5:**
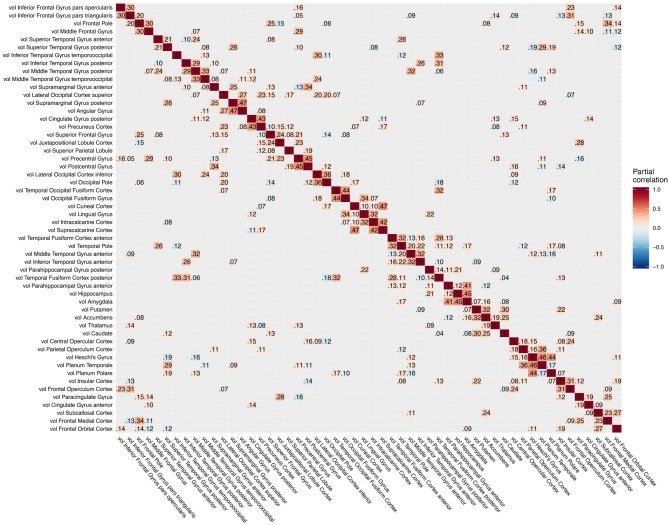
Partial correlation matrix for gray matter volume in the left hemisphere estimated for the combined data of EMCI, LMCI, and AD patients. Averaged over 10 repetitions. Associations of lowest magnitude were not present in all iterations. EMCI/LMCI, early and late amnestic mild cognitive impairment; AD, Alzheimer's dementia; vol, gray matter volume.

For intra-modal associations, i.e., within the same imaging modality, brain regions directly adjacent to each other formed smaller clusters of high partial correlation around the main diagonal (Figures 3–5). When considering inter-modal associations, i.e., between different imaging modalities, we obtained a consistent pattern of significant positive intra-regional conditional dependency for the pairs amyloid-β deposition and metabolism with a mean partial correlation of ρ = 0.21 for 43 significant associations. These are visible as the higher intensities in the diagonal of Figure S2. Between amyloid-β and gray matter volume as well as between metabolism and gray matter volume, only few significant intra-regional associations were found (Figures S3, S4).

### 3.2. Group Comparison of the Graph Structures

**Figure 6:**
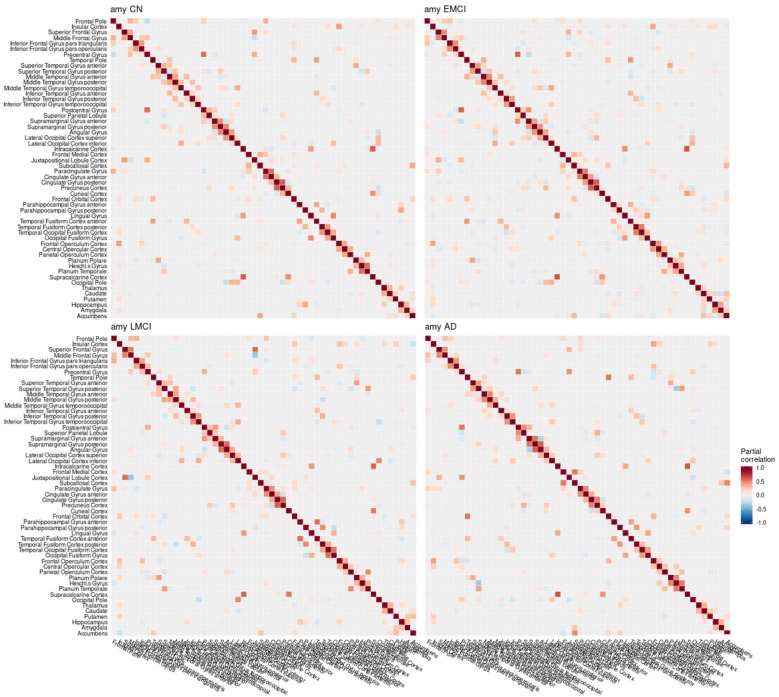
Partial correlation matrix for amyloid-β in the left hemisphere by group. Averaged over 10 repetitions. Associations of lowest magnitude were not present in all iterations. CN, cognitively healthy elderly controls; EMCI/LMCI, early and late amnestic mild cognitive impairment; AD, Alzheimer's dementia; amy, amyloid-β.

**Figure 7:**
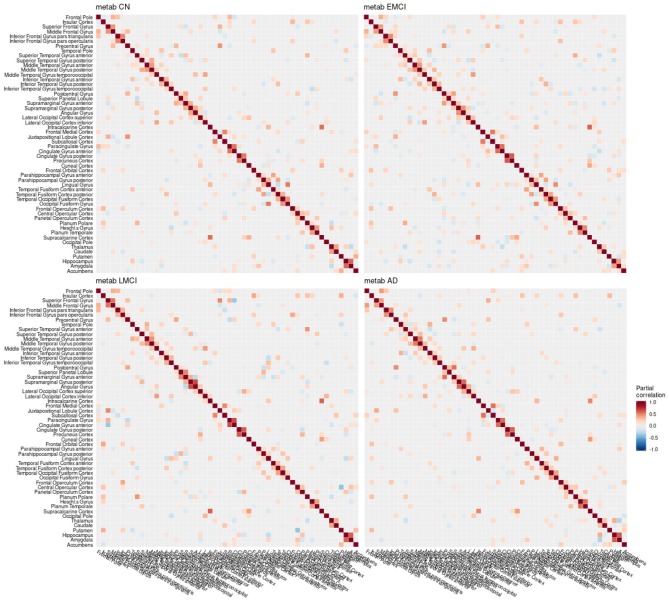
Partial correlation matrix for glucose metabolism in the left hemisphere by group. Averaged over 10 repetitions. Associations of lowest magnitude were not present in all iterations. CN, cognitively healthy elderly controls; EMCI/LMCI, early and late amnestic mild cognitive impairment; AD, Alzheimer's dementia; metab, glucose metabolism.

**Figure 8:**
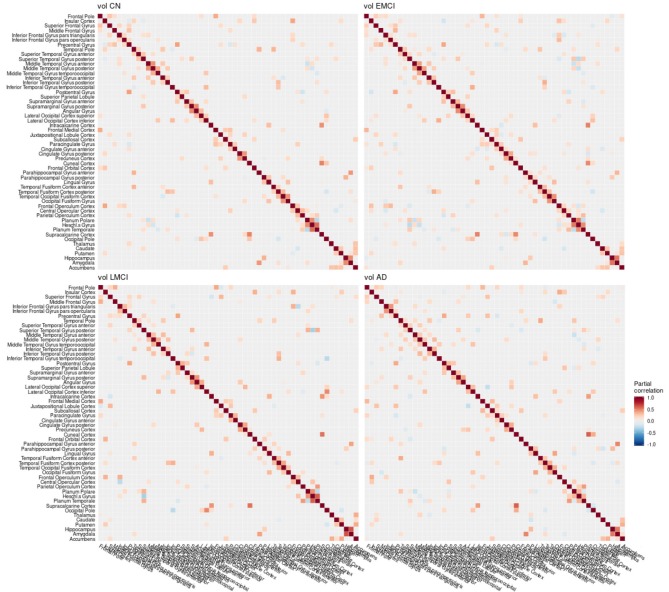
Partial correlation matrix for gray matter volume in the left hemisphere by group. Averaged over 10 repetitions. Associations of lowest magnitude were not present in all iterations. CN, cognitively healthy elderly controls; EMCI/LMCI, early and late amnestic mild cognitive impairment; AD, Alzheimer's dementia; vol, gray matter volume.

**Figure 9:**
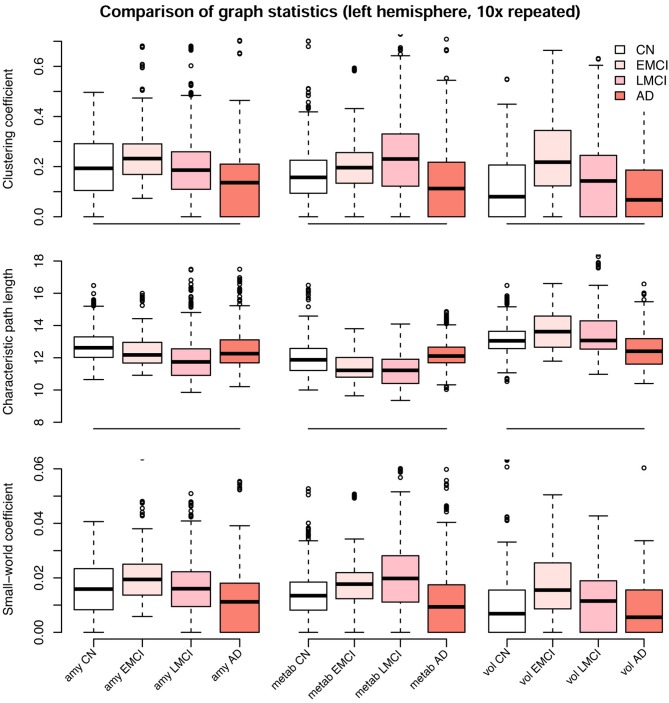
Comparison of graph statistics for the partial correlation matrices of the left hemisphere stratified by diagnostic group and image modality. Estimates based on Gaussian graphical models using multimodal neuroimaging data. The distribution of the weighted clustering coefficient, characteristic weighted path length, and small-world coefficient for individual brain regions is shown. Boxes display median, first and third quartile of the distributions, and whiskers indicate ±1.5 × interquartile range. All blocks showed significant differences in mean between groups, one-way analysis of variance (ANOVA), *df* = 215, *F* > 4, *p* < 0.01. *P*-values for Tukey's honest significant difference tests are given in Table 2. CN, cognitively healthy elderly controls; EMCI/LMCI, early and late amnestic mild cognitive impairment; AD, Alzheimer's dementia; amy, amyloid-β; metab, glucose metabolism; vol, gray matter volume.

When estimating separate models for each diagnostic group based on the multimodal data, graph structures derived from Pearson and partial correlation matrices (Figures 6–8) both differed in their density, leading to significant alterations of the clustering coefficient, characteristic path length, and small-world coefficient (Figure 9 and Figure S9). Detailed graph statistics stratified by individual regions and diagnostic groups are provided in Figures S5–S7.

We observed a biphasic trajectory of the graph measures. This means that the clustering coefficient and small world coefficient initially increases when comparing early MCI and CN participants (Figure 9). When Alzheimer's disease progresses, i.e., in the late MCI and dementia groups, both measures decrease again, with late MCI being approximately on the same level as CN participants (Figure 9). The characteristic path length showed a similar pattern across groups, but with inverted directionality. All blocks showed significant differences in mean between groups, one-way analysis of variance (ANOVA), *df* = 215, *F* ≥ 4, *p* < 0.01, η^2^ ≥ 0.055. Detailed results are provided in Table S2. *P*-values for Tukey's honest significant difference tests are provided in Table 2 and Table S1. Graph statistics obtained from the right hemisphere data (Figure S8) were largely consistent with strongest agreement for the characteristic path length metric.

**Table 2:** *P*-values for the group comparison of partial correlation graph statistics (Figure 9).

		**Amyloid-β**			**Metabolism**			**Volume**		
		**EMCI**	**LMCI**	**AD**	**EMCI**	**LMCI**	**AD**	**EMCI**	**LMCI**	**AD**
Clustering coefficient	CN	0.167	0.999	0.178	0.323	0.021	0.718	0.009	0.977	0.999
	EMCI		0.183	<0.001		0.630	0.031		0.030	0.012
	LMCI			0.162			<0.001			0.990
Path length	CN	0.264	<0.001	0.630	0.015	0.001	0.357	0.106	0.664	0.005
	EMCI		0.189	0.922		0.884	<0.001		0.667	<0.001
	LMCI			0.044			<0.001			<0.001
Small-world coefficient	CN	0.101	0.940	0.301	0.184	0.002	0.701	0.011	0.967	0.987
	EMCI		0.313	<0.001		0.411	0.011		0.042	0.029
	LMCI			0.096			<0.001			0.999

*Adjusted P-values from Tukey's honest significant difference tests, controlling for family-wise error rate within each comparison block. CN, cognitively healthy elderly controls; EMCI/LMCI, early and late amnestic mild cognitive impairment; AD, Alzheimer's dementia*.

## 4. Discussion

### 4.1. Conditional Dependency Between Brain Regions

The GGMs estimated the strongest conditional dependencies mainly *within* imaging modalities. We expected adjacent brain regions to form clusters with high inter-cluster similarity for amyloid-β deposition (Figure 3), as it is known to have low variability in spatial distribution and, therefore, is often used as a dichotomic variable after applying a certain threshold to the global amyloid tracer uptake (Chételat et al., 2013; Landau et al., 2013; Grothe et al., 2017) or as four-stage variable derived from a linear spreading pattern (Grothe et al., 2017; Sakr et al., 2019). We also found such clustering patterns for metabolism (Figure 4) and gray matter volume (Figure 5), matching previous studies on metabolism and gray matter covariance networks based on Pearson correlation (Yao et al., 2010; Carbonell et al., 2016; Pereira et al., 2016) or principal component analysis (Di and Biswal, 2012; Spetsieris et al., 2015; Savio et al., 2017). Clusters of high covariance have been found in the lateral and medial parietal lobe, lateral frontal lobe, and lateral and medial temporal lobe, and had been associated with simultaneous growth during brain development, functional co-activation, and axonal connectivity in the literature (Gong et al., 2012; Alexander-Bloch et al., 2013).

Our analyses yielded only few and relatively weak associations *between* different modalities (Figures S2–S4), except for the direct intra-regional dependency between amyloid-β and metabolism as well as between amyloid and gray matter volume (diagonal of Figures S2, S4), which matched our previous analysis with six selected regions of interest (Dyrba et al., 2017). The positive dependency between amyloid-β and metabolism was strongest in the early MCI group and matches previous results for partial correlation obtained from linear regression models (Altmann et al., 2015). This previous study reported a markedly reduced number and strength of negative associations between regional amyloid-β and metabolism when correcting for global amyloid load. They concluded that the negative association between amyloid deposition and metabolism is more related to the global amyloid level than to the distinct regional level. The pattern of intra-regional dependency between amyloid-β and metabolism as well as between amyloid-β and gray matter volume was strongest in the early MCI group, which could refer to the early phase of the disease and, therefore, a high variation in regional amyloid-β deposition and a strong contribution of the amyloid level on both metabolism and volume (Drzezga et al., 2011; Carbonell et al., 2016). Notably, conditional dependencies between metabolism and volume were obtained only for few regions including hippocampus and putamen, but not for other expected regions such as posterior cingulate cortex (Teipel and Grothe, 2016) (Figure S3).

### 4.2. Alterations of Graph Measures

Various studies reported a network disruption of AD in comparison to cognitively healthy controls for gray matter volume (He et al., 2008; Yao et al., 2010; Li et al., 2012; Tijms et al., 2013; John et al., 2017) and glucose metabolism (Morbelli et al., 2012; Titov et al., 2017), and intermediate levels for volume in MCI (Yao et al., 2010; Pereira et al., 2016); which we could replicate in our sample (Figure S9). However, it has to be noted that for Pearson correlation matrices usually high thresholds are applied to obtain sparser graphs. Chung et al. (2016) and Voevodskaya et al. (2017) reported a high influence of the selected graph density threshold on the graph measures, leading to divergent increases and decreases of the global clustering coefficient metric. To circumvent such problems, we used weighted versions of the graph measures (Rubinov and Sporns, 2010) and proposed GGMs to obtain sparse conditional dependency matrices. Our results suggest that graph statistics for regional dependency networks follow a biphasic trajectory in the course of AD, a pattern that was recently also reported for cortical thinning and mean diffusivity (Montal et al., 2018) and resting-state fMRI connectivity (Schultz et al., 2017).

In the current study, the EMCI group displayed the strongest alterations of network structure with an increase of the clustering coefficient, which may relate to the process of amyloid accumulation taking place in several regions simultaneously in this group increasing the intra-cluster correlation. For amyloid-β and volume, LMCI subjects showed a clustering coefficient and small-world coefficient comparable to controls, in contrast to metabolism, where this group showed strongest deviation from the other groups (Table 2). The lowest alterations of graph measures were obtained for the gray matter network.

GGMs were recently applied as clustering algorithm for brain networks in a few other single-modality applications. de Vos et al. (2017) found them useful for increasing group separation between AD and controls compared to classical Pearson correlation networks in resting-state functional connectivity. Titov et al. (2017) compared metabolic networks for the differential diagnosis between AD and frontotemporal lobar degeneration (FTLD). They also proposed an algorithm to estimate if an individual subject shows a more AD or FTLD pattern of regional metabolism. Munilla et al. (2017) systematically evaluated the influence of the number of subjects and the regularization strength on the GGM stability and graph structure. They found that the estimated GGM graph structure and small-world coefficient converged to a stable level when including 40 or more subjects in their study sample. For regularization-based approximation of GGMs, they showed that the probability of an edge to exist in the estimated graph structure almost linearly corresponds to the magnitude of their partial correlation. Thus, this finding confirms our initial decision, that sampling-based Bayesian estimation of the graph structure might be more useful for detecting even low associations.

### 4.3. Limitations

It has to be noted that our methodological framework can currently only be applied as a group statistic but not for individual subjects. Therefore, GGMs can be used for exploratory analyses as alternative to Pearson correlation networks, and may aid generating new hypotheses about the interrelation of clinical variables or feature selection. Then, derived hypotheses can be validated using classical statistical methods such as regression or mediation analysis.

Another limitation is the high uncertainty in the statistical model to estimate the partial correlations. This is due to the theoretically hard problem of matrix inversion on the one hand, and due to the high number of possible graph edges in comparison to the sample size on the other hand. Thus, the model might be fragile with respect to the obtained values and requires large training samples to get stable results. Here, we repeated the model estimation on the whole data for ten times to observe the effect on model stability, which was yielding largely consistent results for strong links with high partial correlation, but getting more variable for weaker links with low partial correlation. Replicating the results using the right hemisphere data also yielded largely consistent results with highest agreement for the characteristic path length metric. Apparent deviation in clustering coefficient and consequently in small-world coefficient (= ratio of both) might be explained by the asymmetry of the brain and the lateralization reported for Alzheimer's disease in the literature (e.g., stronger left hippocampus atrophy in ADNI) (Grothe and Teipel, 2016; Wei, 2018). However, our findings still need to be replicated in independent cohorts.

We observed a saturation of the conditional dependency network when adding many variables. This means, the model parameters might strongly change when having only few variables in the model and adding another variable; in contrast to very stable estimates of larger models with dozens of variables, which are hardly altered when adding another variable. Actually, this problem is well-known for linear regression models and related to multicollinearity in the data (O'brien, 2007; Dormann et al., 2013; Teipel S. J. et al., 2015). Recent developments in stochastic block models may help to overcome these limitations, as they try to infer the underlying clustering block structure and separately estimate statistical associations within and between clusters (Sun et al., 2014; Hosseini and Lee, 2016).

### 4.4. Conclusion

We applied GGMs to assess inter-modal and inter-regional dependencies of high-dimensional multimodal neuroimaging data of AD-related brain alterations. Our results showed that conditional dependency networks estimated by GGMs provide useful information within imaging modalities and could be used as alternative to Pearson-correlation networks. Nonetheless, GGMs did not detect some expected associations between modalities and, therefore, may have limited applicability for large-scale data with dozens of variables.

## Data Availability Statement

MRI and PET data being used in this study can be retrieved from ADNI (http://adni.loni.usc.edu/data-samples/access-data/). Processed imaging data and extracted regional mean values are available from the corresponding authors upon request. The R package BDgraph can be downloaded from CRAN (https://cran.r-project.org/web/packages/BDgraph) or GitHub (https://github.com/cran/BDgraph).

## Ethics Statement

The studies involving human participants were reviewed and approved by ADNI internal review board. The patients/participants provided their written informed consent to participate in this study.

## Author Contributions

MD, RM, TK, and ST designed the study. MG and MD preprocessed the imaging data. MD and RM conducted the statistical analyses. MG, TK, and ST aided in interpreting the results. MD drafted the first version of the manuscript. All authors revised the manuscript and contributed to the final version.

## Conflict of Interest

The authors declare that the research was conducted in the absence of any commercial or financial relationships that could be construed as a potential conflict of interest.

## Footnotes

^1^For Markov chain Monte Carlo (MCMC) methods, burn-in refers to the practice of discarding an initial portion of the Markov chain sample, so that the chain can reach a stationary distribution. Thus, the effect of randomly chosen initial values on the posterior inference is minimized.

## References

[B1] (2018) Left lateralized cerebral glucose metabolism declines in amyloid-beta positive persons with mild cognitive impairment. NeuroImage Clin. 20, 286–296. 10.1016/j.nicl.2018.07.016 PMC6084012 30101060

[B2] Alexander-Bloch A. Raznahan A. Bullmore E. Giedd J. (2013). The convergence of maturational change and structural covariance in human cortical networks. J. Neurosci. 33, 2889–2899. 10.1523/JNEUROSCI.3554-12.2013 23407947 PMC3711653

[B3] Altmann A. Ng B. Landau S. M. Jagust W. J. Greicius M. D. (2015). Regional brain hypometabolism is unrelated to regional amyloid plaque burden. Brain 138(Pt 12), 3734–3746. 10.1093/brain/awv278 26419799 PMC4806718

[B4] Bontempi G. Flauder M. (2015). From dependency to causality: a machine learning approach. J. Mach. Learn. Res. 16, 2437–2457. 10.5555/2789272.2912076

[B5] Buckner R. L. Sepulcre J. Talukdar T. Krienen F. M. Liu H. Hedden T.. (2009). Cortical hubs revealed by intrinsic functional connectivity: mapping, assessment of stability, and relation to Alzheimer's disease. J. Neurosci. 29, 1860–1873. 10.1523/JNEUROSCI.5062-08.2009 19211893 PMC2750039

[B6] Buckner R. L. Snyder A. Z. Shannon B. J. LaRossa G. Sachs R. Fotenos A. F.. (2005). Molecular, structural, and functional characterization of Alzheimer's disease: evidence for a relationship between default activity, amyloid, and memory. J. Neurosci. 25, 7709–7717. 10.1523/JNEUROSCI.2177-05.2005 16120771 PMC6725245

[B7] Cai T. T. Li H. Liu W. Xie J. (2013). Covariate-adjusted precision matrix estimation with an application in genetical genomics. Biometrika 100, 139–156. 10.1093/biomet/ass058 28316337 PMC5351557

[B8] Carbonell F. Zijdenbos A. P. McLaren D. G. Iturria-Medina Y. Bedell B. J. (2016). Modulation of glucose metabolism and metabolic connectivity by beta-amyloid. J. Cereb. Blood Flow Metab. 36, 2058–2071. 10.1177/0271678X16654492 27301477 PMC5363668

[B9] Chang Y.-T. Huang C.-W. Chang Y.-H. Chen N.-C. Lin K.-J. Yan T.-C.. (2015). Amyloid burden in the hippocampus and default mode network: relationships with gray matter volume and cognitive performance in mild stage Alzheimer disease. Medicine 94:e763. 10.1097/MD.0000000000000763 25906109 PMC4602683

[B10] Chételat G. La Joie R. Villain N. Perrotin A. de La Sayette V. Eustache F.. (2013). Amyloid imaging in cognitively normal individuals, at-risk populations and preclinical Alzheimer's disease. NeuroImage Clin. 2, 356–365. 10.1016/j.nicl.2013.02.006 24179789 PMC3777672

[B11] Chung J. Yoo K. Kim E. Na D. L. Jeong Y. (2016). Glucose metabolic brain networks in early-onset vs. late-onset Alzheimer's disease. Front. Aging Neurosci. 8:159. 10.3389/fnagi.2016.00159 27445800 PMC4928512

[B12] de Vos F. Koini M. Schouten T. M. Seiler S. van der Grond J. Lechner A.. (2017). A comprehensive analysis of resting state fmri measures to classify individual patients with Alzheimer's disease. NeuroImage 167, 62–72. 10.1016/j.neuroimage.2017.11.025 29155080

[B13] Desikan R. S. Ségonne F. Fischl B. Quinn B. T. Dickerson B. C. Blacker D.. (2006). An automated labeling system for subdividing the human cerebral cortex on MRI scans into gyral based regions of interest. NeuroImage 31, 968–980. 10.1016/j.neuroimage.2006.01.021 16530430

[B14] Di X. Biswal B. B. (2012). Metabolic brain covariant networks as revealed by FDG-PET with reference to resting-state fMRI networks. Brain Connect. 2, 275–283. 10.1089/brain.2012.0086 23025619 PMC3621675

[B15] Dormann C. F. Elith J. Bacher S. Buchmann C. Carl G. Carré G. Marquéz J. R. G. (2013). Collinearity: a review of methods to deal with it and a simulation study evaluating their performance. Ecography 36, 27–46. 10.1111/j.1600-0587.2012.07348.x

[B16] Drzezga A. Becker J. A. van Dijk K. R. A. Sreenivasan A. Talukdar T. Sullivan C.. (2011). Neuronal dysfunction and disconnection of cortical hubs in non-demented subjects with elevated amyloid burden. Brain 134(Pt 6), 1635–1646. 10.1093/brain/awr066 21490054 PMC3102239

[B17] Dyrba M. Grothe M. J. Mohammadi A. Binder H. Kirste T. Teipel S. J. (2017). Comparison of different hypotheses regarding the spread of Alzheimer's disease using Markov random fields and multimodal imaging. J. Alzheimer's Dis. 65, 731–746. 10.3233/JAD-161197 28697557

[B18] Gong G. He Y. Chen Z. J. Evans A. C. (2012). Convergence and divergence of thickness correlations with diffusion connections across the human cerebral cortex. NeuroImage 59, 1239–1248. 10.1016/j.neuroimage.2011.08.017 21884805

[B19] Gonzalez-Escamilla G. Lange C. Teipel S. Buchert R. Grothe M. J. (2017). PETPVE12: an SPM toolbox for partial volume effects correction in brain PET – application to amyloid imaging with AV45-PET. NeuroImage 147, 669–677. 10.1016/j.neuroimage.2016.12.077 28039094

[B20] Grothe M. Heinsen H. Teipel S. (2013). Longitudinal measures of cholinergic forebrain atrophy in the transition from healthy aging to alzheimer's disease. Neurobiol. Aging 34, 1210–1220. 10.1016/j.neurobiolaging.2012.10.018 23158764 PMC4058576

[B21] Grothe M. J. Barthel H. Sepulcre J. Dyrba M. Sabri O. Teipel S. J. (2017). *In vivo* staging of regional amyloid deposition. Neurology 89, 2031–2038. 10.1212/WNL.0000000000004643 29046362 PMC5711511

[B22] Grothe M. J. Heinsen H. Amaro E. Grinberg L. T. Teipel S. J. (2016). Cognitive correlates of basal forebrain atrophy and associated cortical hypometabolism in mild cognitive impairment. Cereb. Cortex 26, 2411–2426. 10.1093/cercor/bhv062 25840425 PMC4869802

[B23] Grothe M. J. Teipel S. J. (2016). Spatial patterns of atrophy, hypometabolism, and amyloid deposition in Alzheimer's disease correspond to dissociable functional brain networks. Hum. Brain Mapp. 37, 35–53. 10.1002/hbm.23018 26441321 PMC4715545

[B24] Hastie T. J. Tibshirani R. J. Friedman J. H. (2013). The Elements of Statistical Learning: Data Mining, Inference, and Prediction, 2nd Edn. Springer Series in Statistics. New York, NY: Springer.

[B25] He Y. Chen Z. Evans A. (2008). Structural insights into aberrant topological patterns of large-scale cortical networks in alzheimer's disease. J. Neurosci. 28, 4756–4766. 10.1523/JNEUROSCI.0141-08.2008 18448652 PMC6670444

[B26] Hlinka J. Hartman D. Jajcay N. Tomeček D. Tintěra J. Paluš M. (2017). Small-world bias of correlation networks: from brain to climate. Chaos 27:035812. 10.1063/1.4977951 28364746

[B27] Hosseini M. J. Lee S.-I. (2016). Learning sparse gaussian graphical models with overlapping blocks, in Advances in Neural Information Processing Systems 29, eds D. D. Lee, M. Sugiyama, U. V. Luxburg, I. Guyon, and R. Garnett (Red Hook, NY: Curran Associates, Inc.), 3808–3816.

[B28] Iturria-Medina Y. Carbonell F. M. Sotero R. C. Chouinard-Decorte F. Evans A. C. (2017). Multifactorial causal model of brain (dis)organization and therapeutic intervention: application to Alzheimer's disease. NeuroImage 152, 60–77. 10.1016/j.neuroimage.2017.02.058 28257929

[B29] John M. Ikuta T. Ferbinteanu J. (2017). Graph analysis of structural brain networks in Alzheimer's disease: beyond small world properties. Brain Struct. Funct. 222, 923–942. 10.1007/s00429-016-1255-4 27357309

[B30] Kljajevic V. Grothe M. J. Ewers M. Teipel S. (2014). Distinct pattern of hypometabolism and atrophy in preclinical and predementia Alzheimer's disease. Neurobiol. Aging 35, 1973–1981. 10.1016/j.neurobiolaging.2014.04.006 24811241

[B31] Koller D. Friedman N. (2009). Probabilistic Graphical Models: Principles and Techniques. Adaptive Computation and Machine Learning. Cambridge, MA: MIT Press.

[B32] La Joie R. Perrotin A. Barré L. Hommet C. Mézenge F. Ibazizene M.. (2012). Region-specific hierarchy between atrophy, hypometabolism, and β-amyloid (aβ) load in Alzheimer's disease dementia. J. Neurosci. 32, 16265–16273. 10.1523/JNEUROSCI.2170-12.2012 23152610 PMC6794030

[B33] Landau S. M. Breault C. Joshi A. D. Pontecorvo M. Mathis C. A. Jagust W. J.. (2013). Amyloid-beta imaging with pittsburgh compound b and florbetapir: comparing radiotracers and quantification methods. J. Nucl. Med. 54, 70–77. 10.2967/jnumed.112.109009 23166389 PMC3747730

[B34] Lauritzen S. L. (1996). Graphical Models, Vol. 17. Oxford Statistical Science Series. Oxford: Clarendon Press.

[B35] Li Y. Wang Y. Wu G. Shi F. Zhou L. Lin W. Shen D. (2012). Discriminant analysis of longitudinal cortical thickness changes in Alzheimer's disease using dynamic and network features. Neurobiol. Aging 33, 427.e15–30. 10.1016/j.neurobiolaging.2010.11.008 21272960 PMC3086988

[B36] Mårtensson G. Pereira J. B. Mecocci P. Vellas B. Tsolaki M. Kłoszewska I.. (2018). Stability of graph theoretical measures in structural brain networks in Alzheimer's disease. Sci. Rep. 8:11592. 10.1038/s41598-018-29927-0 30072774 PMC6072788

[B37] Madigan D. Raftery A. E. Volinsky C. Hoeting J. (1996). Bayesian model averaging, in Proceedings of the AAAI Workshop on Integrating Multiple Learned Models (Portland, OR), 77–83.

[B38] Meinshausen N. Bühlmann P. (2006). High-dimensional graphs and variable selection with the lasso. Ann. Stat. 34, 1436–1462. 10.1214/009053606000000281

[B39] Mohammadi A. Wit E. C. (2015). Bayesian structure learning in sparse Gaussian graphical models. Bayesian Anal. 10, 109–138. 10.1214/14-BA889

[B40] Mohammadi R. Wit E. C. (2019). BDgraph: an R package for Bayesian structure learning in graphical models. J. Stat. Soft. 89, 1–30. 10.18637/jss.v089.i03

[B41] Montal V. Vilaplana E. Alcolea D. Pegueroles J. Pasternak O. Gonz?lez-Ortiz S.. (2018). Cortical microstructural changes along the Alzheimer's disease continuum. Alzheimer's Dement. 14, 340–351. 10.1016/j.jalz.2017.09.013 29080407

[B42] Morbelli S. Drzezga A. Perneczky R. Frisoni G. B. Caroli A. van Berckel B. N. M.. (2012). Resting metabolic connectivity in prodromal Alzheimer's disease. A European Alzheimer disease consortium (EADC) project. Neurobiol. Aging 33, 2533–2550. 10.1016/j.neurobiolaging.2012.01.005 22365486

[B43] Müller-Gärtner H. W. Links J. M. Prince J. L. Bryan R. N. McVeigh E. Leal J. P.. (1992). Measurement of radiotracer concentration in brain gray matter using positron emission tomography: MRI-based correction for partial volume effects. J. Cereb. Blood Flow Metab. 12, 571–583. 10.1038/jcbfm.1992.81 1618936

[B44] Munilla J. Ortiz A. Górriz J. M. Ramírez J. (2017). Construction and analysis of weighted brain networks from sice for the study of Alzheimer's disease. Front. Neuroinform. 11:19. 10.3389/fninf.2017.00019 28344551 PMC5344925

[B45] O'brien R. M. (2007). A caution regarding rules of thumb for variance inflation factors. Qual. Quant. 41, 673–690. 10.1007/s11135-006-9018-6

[B46] Onnela J.-P. Saramäki J. Kertész J. Kaski K. (2005). Intensity and coherence of motifs in weighted complex networks. Phys. Rev. E 71:065103. 10.1103/PhysRevE.71.065103 16089800

[B47] Pereira J. B. Mijalkov M. Kakaei E. Mecocci P. Vellas B. Tsolaki M.. (2016). Disrupted network topology in patients with stable and progressive mild cognitive impairment and Alzheimer's disease. Cereb. Cortex 26, 3476–3493. 10.1093/cercor/bhw128 27178195 PMC4961019

[B48] Ravikumar P. Wainwright M. J. Raskutti G. Yu B. (2011). High-dimensional covariance estimation by minimizing l1-penalized log-determinant divergence. Electron. J. Stat. 5, 935–980. 10.1214/11-EJS631

[B49] Rubinov M. Sporns O. (2010). Complex network measures of brain connectivity: uses and interpretations. NeuroImage 52, 1059–1069. 10.1016/j.neuroimage.2009.10.003 19819337

[B50] Ryali S. Chen T. Supekar K. Menon V. (2012). Estimation of functional connectivity in fmri data using stability selection-based sparse partial correlation with elastic net penalty. NeuroImage 59, 3852–3861. 10.1016/j.neuroimage.2011.11.054 22155039 PMC3288428

[B51] Sakr F. A. Grothe M. J. Cavedo E. Jelistratova I. Habert M.-O. Dyrba M.. (2019). Applicability of *in vivo* staging of regional amyloid burden in a cognitively normal cohort with subjective memory complaints: the INSIGHT-preAD study. Alzheimer's Res. Ther. 11:15. 10.1186/s13195-019-0466-3 30704537 PMC6357385

[B52] Savio A. Fünger S. Tahmasian M. Rachakonda S. Manoliu A. Sorg C.. (2017). Resting-state networks as simultaneously measured with functional MRI and PET. J. Nucl. Med. 58, 1314–1317. 10.2967/jnumed.116.185835 28254868 PMC6944183

[B53] Schultz A. P. Chhatwal J. P. Hedden T. Mormino E. C. Hanseeuw B. J. Sepulcre J.. (2017). Phases of hyperconnectivity and hypoconnectivity in the default mode and salience networks track with amyloid and tau in clinically normal individuals. J. Neurosci. 37, 4323–4331. 10.1523/JNEUROSCI.3263-16.2017 28314821 PMC5413178

[B54] Seeley W. W. Crawford R. K. Zhou J. Miller B. L. Greicius M. D. (2009). Neurodegenerative diseases target large-scale human brain networks. Neuron 62, 42–52. 10.1016/j.neuron.2009.03.024 19376066 PMC2691647

[B55] Sepulcre J. Sabuncu M. R. Becker A. Sperling R. Johnson K. A. (2013). *In vivo* characterization of the early states of the amyloid-beta network. Brain 136(Pt 7), 2239–2252. 10.1093/brain/awt146 23801740 PMC3692037

[B56] Sepulcre J. Sabuncu M. R. Li Q. El Fakhri G. Sperling R. Johnson K. A. (2017). Tau and amyloid β proteins distinctively associate to functional network changes in the aging brain. Alzheimer's Dement. 13, 1261–1269. 10.1016/j.jalz.2017.02.011 28366797 PMC5623176

[B57] Spetsieris P. G. Ko J. H. Tang C. C. Nazem A. Sako W. Peng S.. (2015). Metabolic resting-state brain networks in health and disease. Proc. Natl. Acad. Sci. U.S.A. 112, 2563–2568. 10.1073/pnas.1411011112 25675473 PMC4345616

[B58] Stam C. Jones B. Nolte G. Breakspear M. Scheltens P. (2006). Small-world networks and functional connectivity in Alzheimer's disease. Cereb. Cortex 17, 92–99. 10.1093/cercor/bhj127 16452642

[B59] Sun S. Zhu Y. Xu J. (2014). Adaptive variable clustering in Gaussian graphical models, in Proceedings of the Seventeenth International Conference on Artificial Intelligence and Statistics, Vol. 33 of *Proceedings of Machine Learning Research*, eds S. Kaski and J. Corander (Iceland: Reykjavik), 931–939.

[B60] Teipel S. Drzezga A. Grothe M. J. Barthel H. Chételat G. Schuff N.. (2015). Multimodal imaging in Alzheimer's disease: validity and usefulness for early detection. Lancet Neurol. 14, 1037–1053. 10.1016/S1474-4422(15)00093-9 26318837

[B61] Teipel S. Grothe M. J. (2016). Does posterior cingulate hypometabolism result from disconnection or local pathology across preclinical and clinical stages of Alzheimer's disease? Eur. J. Nucl. Med. Mol. Imaging 43, 526–536. 10.1007/s00259-015-3222-3 26555082 PMC6166099

[B62] Teipel S. Grothe M. J. Zhou J. Sepulcre J. Dyrba M. Sorg C.. (2016). Measuring cortical connectivity in Alzheimer's disease as a brain neural network pathology: toward clinical applications. J. Int. Neuropsychol. Soc. 22, 138–163. 10.1017/S1355617715000995 26888613

[B63] Teipel S. J. Kurth J. Krause B. Grothe M. J. (2015). The relative importance of imaging markers for the prediction of Alzheimer's disease dementia in mild cognitive impairment - beyond classical regression. NeuroImage Clin. 8, 583–593. 10.1016/j.nicl.2015.05.006 26199870 PMC4506984

[B64] Tijms B. M. Möller C. Vrenken H. Wink A. M. de Haan W. van der Flier W. M.. (2013). Single-subject grey matter graphs in Alzheimer's disease. PLoS ONE 8:e58921. 10.1371/journal.pone.0058921 23536835 PMC3594199

[B65] Titov D. Diehl-Schmid J. Shi K. Perneczky R. Zou N. Grimmer T.. (2017). Metabolic connectivity for differential diagnosis of dementing disorders. J. Cereb. Blood Flow Metab. 37, 252–262. 10.1177/0271678X15622465 26721391 PMC5363743

[B66] Torok J. Maia P. D. Powell F. Pandya S. Raj A. (2018). A method for inferring regional origins of neurodegeneration. Brain 141, 863–876. 10.1093/brain/awx371 29409009 PMC5837438

[B67] Villain N. Fouquet M. Baron J.-C. Mézenge F. Landeau B. de La Sayette V.. (2010). Sequential relationships between grey matter and white matter atrophy and brain metabolic abnormalities in early Alzheimer's disease. Brain 133, 3301–3314. 10.1093/brain/awq203 20688814 PMC3291528

[B68] Voevodskaya O. Pereira J. B. Volpe G. Lindberg O. Stomrud E. van Westen D.. (2017). Altered structural network organization in cognitively normal individuals with amyloid pathology. Neurobiol. Aging 64, 15–24. 10.1016/j.neurobiolaging.2017.11.014 29316528

[B69] Wang Y. Kang J. Kemmer P. B. Guo Y. (2016). An efficient and reliable statistical method for estimating functional connectivity in large scale brain networks using partial correlation. Front. Neurosci. 10:123. 10.3389/fnins.2016.00123 27242395 PMC4876368

[B70] Watts D. J. Strogatz S. H. (1998). Collective dynamics of 'small-world' networks. Nature 393, 440–442. 10.1038/30918 9623998

[B71] Yao Z. Zhang Y. Lin L. Zhou Y. Xu C. Jiang T. (2010). Abnormal cortical networks in mild cognitive impairment and Alzheimer's disease. PLoS Comput. Biol. 6:e1001006. 10.1371/journal.pcbi.1001006 21124954 PMC2987916

[B72] Zhou J. Gennatas E. D. Kramer J. H. Miller B. L. Seeley W. W. (2012). Predicting regional neurodegeneration from the healthy brain functional connectome. Neuron 73, 1216–1227. 10.1016/j.neuron.2012.03.004 22445348 PMC3361461

