# Supplementary material for: Gaussian Graphical Models Reveal Inter-Modal and Inter-Regional Conditional Dependencies of Brain Alterations in Alzheimer's Disease
Source: Front Aging Neurosci. 2020 Apr 21;12:99. doi: 10.3389/fnagi.2020.00099 (PMC7186311; doi:10.3389/fnagi.2020.00099)
Supplement: Supplementary file 1 [file Data_Sheet_1.pdf]

# Supplementary material for Dyrba et al. (2020) Gaussian graphical models reveal inter-modal and inter-regional conditional dependencies of brain alterations in Alzheimer's disease.

Frontiers in Aging Neuroscience | doi: 10.3389/fnagi.2020.00099

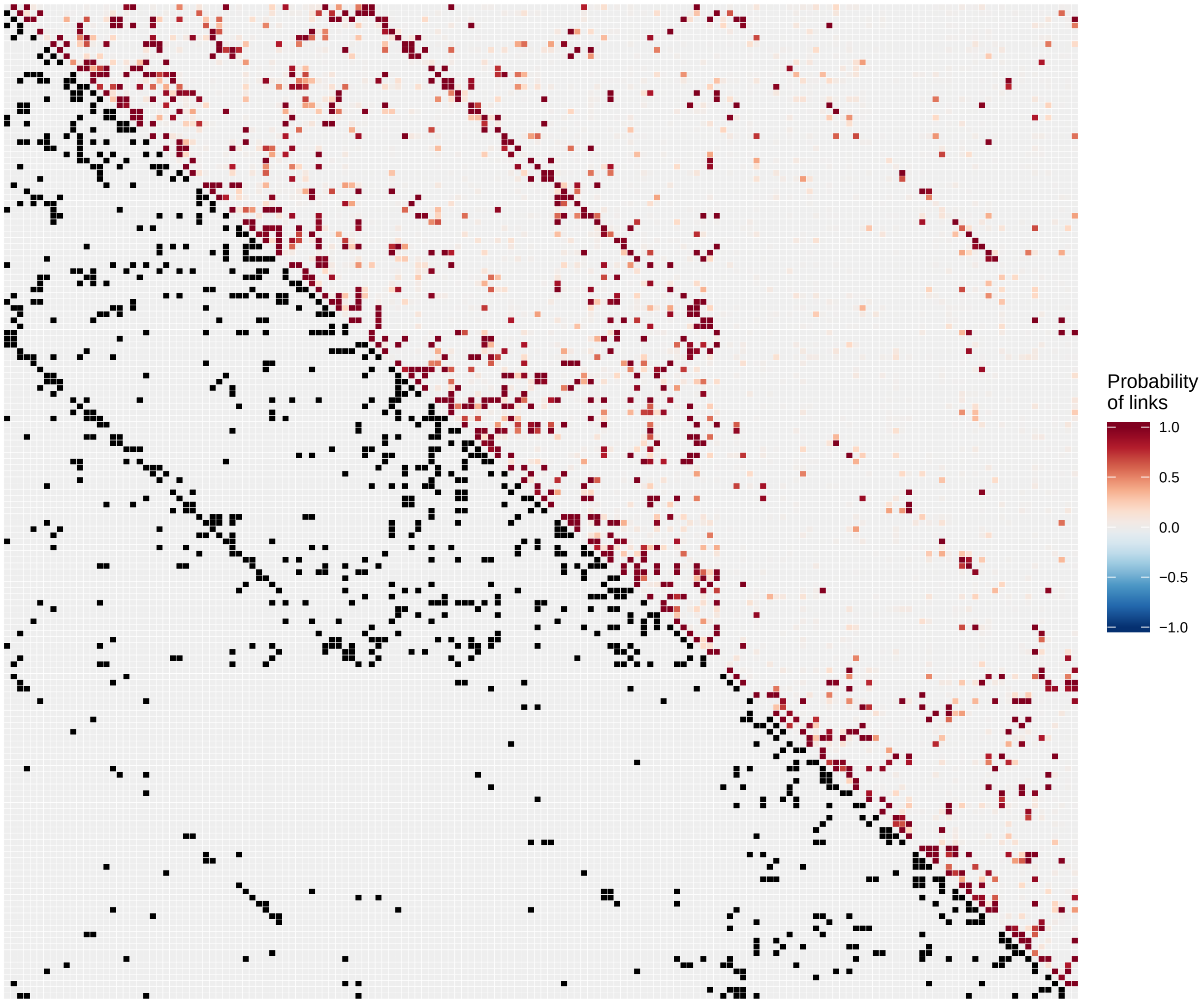


**Figure S1.** Probability of estimated edges for the left hemisphere. The upper right part provides the raw probability of each edge to exist. The lower left part indicates the selected edges exceeding the threshold of $P_{avg} > 0.5$.

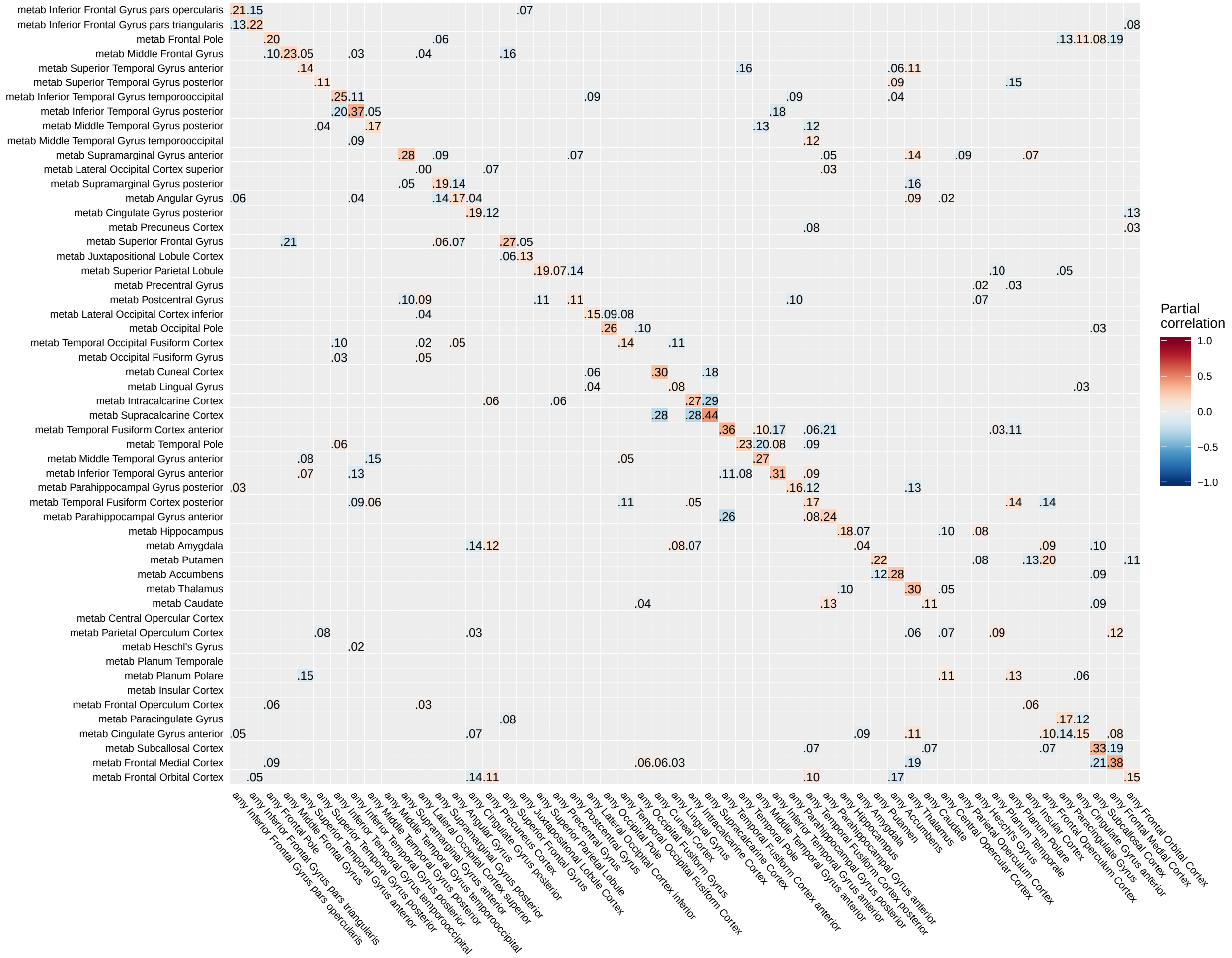


**Figure S2.** Partial correlation matrix for amyloid-$\beta$ deposition and glucose metabolism in the left hemisphere estimated for the combined data of EMCI, LMCI and AD patients. Averaged over ten repetitions. Associations of lowest magnitude were not present in all iterations.
EMCI/LMCI: early and late amnestic mild cognitive impairment, AD: Alzheimer's dementia, amy: amyloid-$\beta$, metab: glucose metabolism.

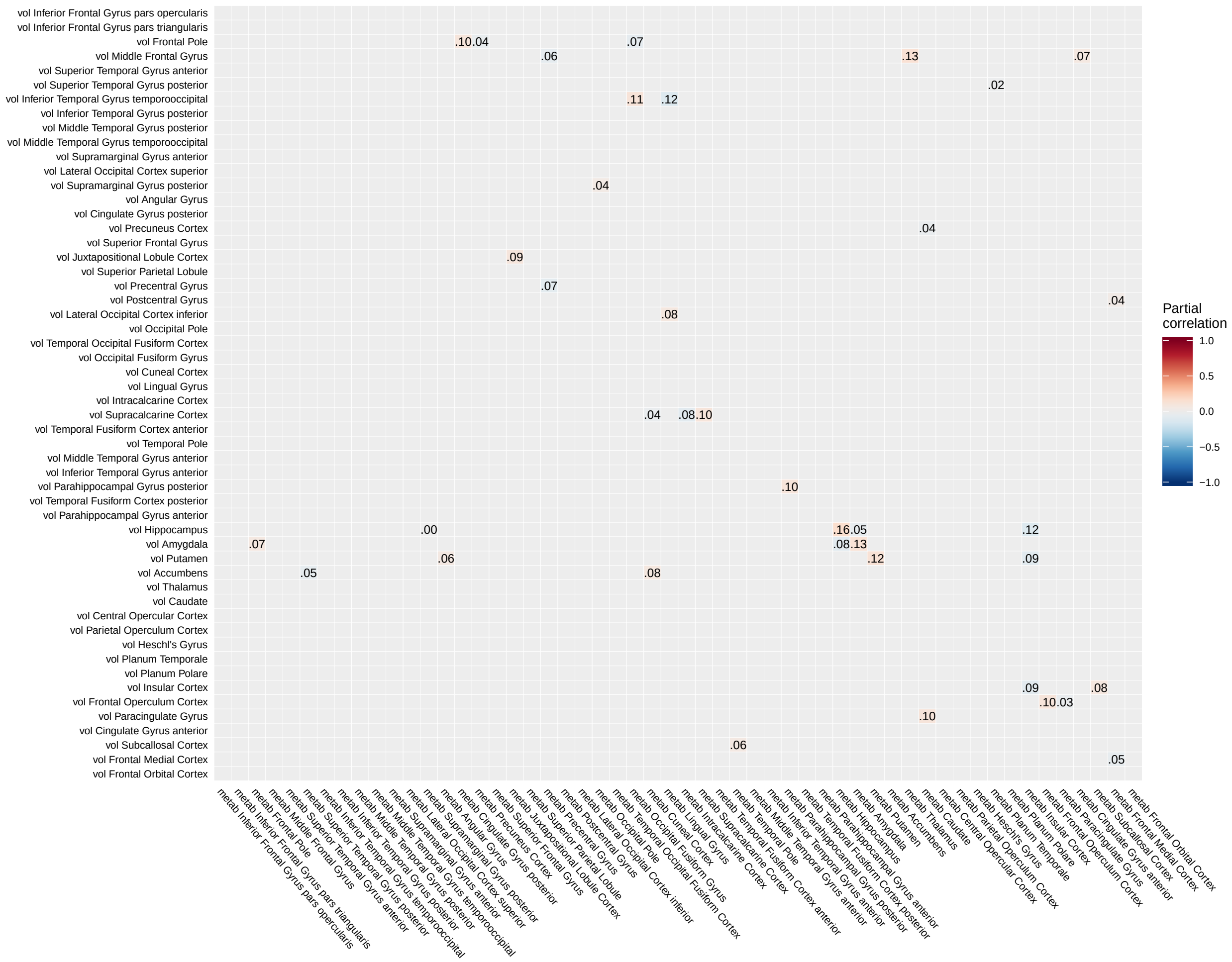

**Figure S3.** Partial correlation matrix for glucose metabolism and gray matter volume in the left hemisphere estimated for the combined data of EMCI, LMCI and AD patients. Averaged over ten repetitions. Associations of lowest magnitude were not present in all iterations.
EMCI/LMCI: early and late amnestic mild cognitive impairment, AD: Alzheimer's dementia, metab: glucose metabolism, vol: gray matter volume.

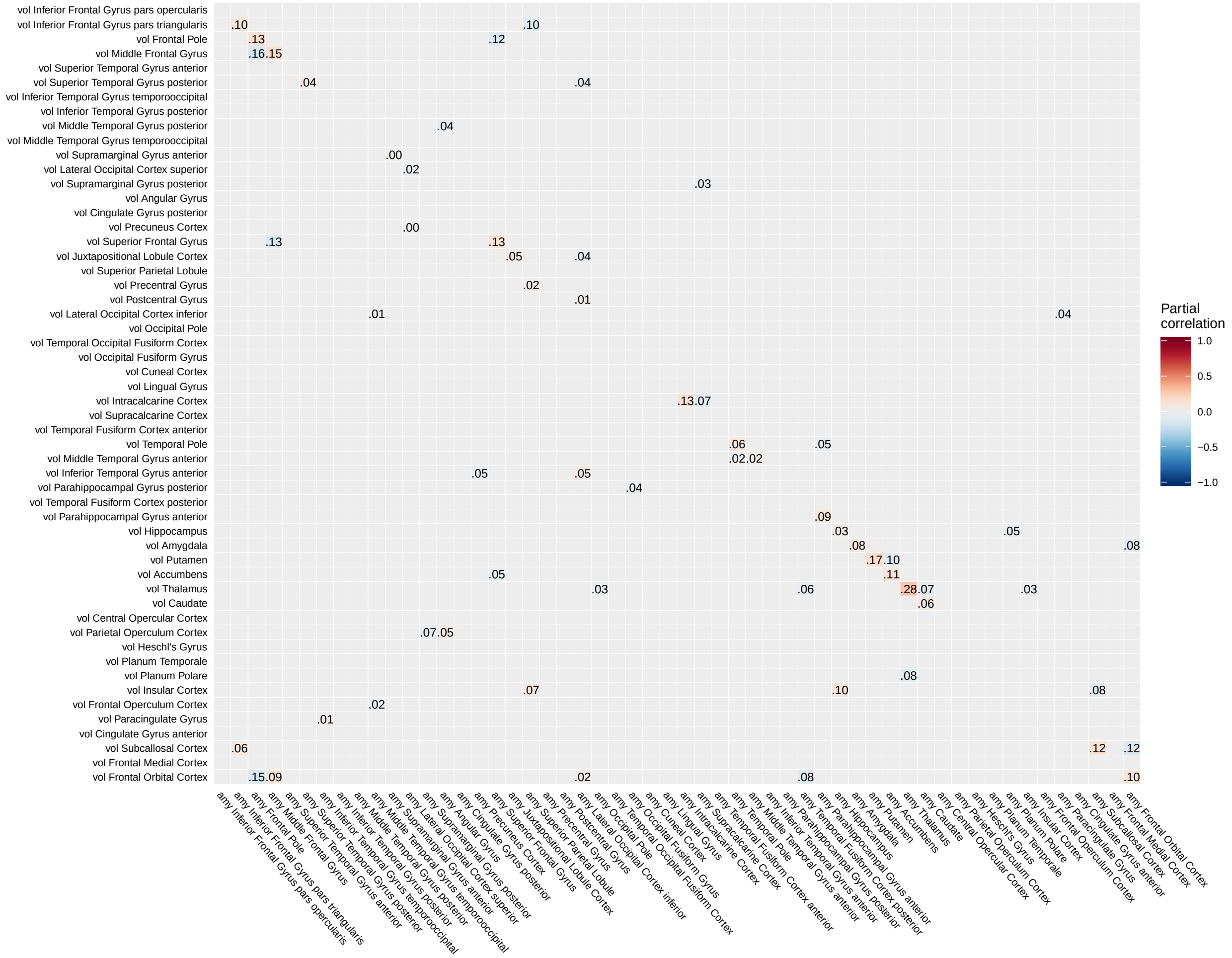

**Figure S4.** Partial correlation matrix for amyloid-$\beta$ deposition and gray matter volume in the left hemisphere estimated for the combined data of EMCI, LMCI and AD patients. Averaged over ten repetitions. Associations of lowest magnitude were not present in all iterations.
EMCI/LMCI: early and late amnestic mild cognitive impairment, AD: Alzheimer's dementia, amy: amyloid-$\beta$, vol: gray matter volume.

Weighted clustering coefficient by region

| | amy CN | amy EMCI | amy LMCI | amy AD | metab CN | metab EMCI | metab LMCI | metab AD | vol CN | vol EMCI | vol LMCI | vol AD |
|---|---|---|---|---|---|---|---|---|---|---|---|---|
| Inferior Frontal Gyrus pars opercularis | .16 | .18 | .12 | .12 | .14 | .02 | .04 | .26 | .19 | .32 | .00 | .00 |
| Inferior Frontal Gyrus pars triangularis | .20 | .08 | .30 | .00 | .00 | .09 | .18 | .00 | .11 | .22 | .17 | .00 |
| Frontal Pole | .21 | .16 | .25 | .11 | .16 | .25 | .35 | .01 | .00 | .08 | .23 | .08 |
| Middle Frontal Gyrus | .29 | .28 | .24 | .11 | .14 | .18 | .24 | .00 | .37 | .66 | .03 | .10 |
| Superior Temporal Gyrus anterior | .12 | .19 | .07 | .31 | .24 | .14 | .08 | .15 | .00 | .07 | .00 | .00 |
| Superior Temporal Gyrus posterior | .17 | .20 | .15 | .14 | .14 | .19 | .21 | .14 | .03 | .34 | .33 | .10 |
| Inferior Temporal Gyrus temporooccipital | .12 | .18 | .17 | .22 | .09 | .14 | .22 | .06 | .00 | .59 | .23 | .00 |
| Inferior Temporal Gyrus posterior | .00 | .17 | .29 | .25 | .10 | .19 | .14 | .08 | .00 | .23 | .15 | .00 |
| Middle Temporal Gyrus posterior | .17 | .11 | .22 | .19 | .00 | .12 | .13 | .17 | .20 | .21 | .01 | .02 |
| Middle Temporal Gyrus temporooccipital | .24 | .28 | .14 | .00 | .23 | .59 | .47 | .55 | .00 | .00 | .00 | .03 |
| Supramarginal Gyrus anterior | .25 | .31 | .30 | .20 | .31 | .18 | .16 | .18 | .12 | .45 | .15 | .00 |
| Lateral Occipital Cortex superior | .41 | .43 | .13 | .20 | .15 | .18 | .25 | .16 | .29 | .08 | .00 | .27 |
| Supramarginal Gyrus posterior | .39 | .18 | .02 | .40 | .26 | .24 | .46 | .09 | .00 | .23 | .13 | .00 |
| Angular Gyrus | .35 | .33 | .13 | .21 | .24 | .12 | .46 | .14 | .15 | .00 | .00 | .06 |
| Cingulate Gyrus posterior | .31 | .32 | .50 | .33 | .12 | .16 | .44 | .33 | .00 | .14 | .02 | .00 |
| Precuneus Cortex | .33 | .29 | .16 | .20 | .08 | .33 | .52 | .25 | .00 | .21 | .00 | .09 |
| Superior Frontal Gyrus | .37 | .15 | .34 | .00 | .19 | .31 | .39 | .00 | .16 | .20 | .10 | .08 |
| Juxtapositional Lobule Cortex | .24 | .13 | .27 | .19 | .18 | .22 | .26 | .01 | .30 | .39 | .40 | .31 |
| Superior Parietal Lobule | .49 | .19 | .40 | .17 | .44 | .28 | .54 | .22 | .00 | .00 | .00 | .25 |
| Precentral Gyrus | .37 | .28 | .14 | .19 | .10 | .21 | .30 | .26 | .11 | .18 | .00 | .18 |
| Postcentral Gyrus | .30 | .25 | .21 | .37 | .13 | .27 | .17 | .35 | .20 | .12 | .00 | .03 |
| Lateral Occipital Cortex inferior | .28 | .41 | .24 | .11 | .17 | .36 | .33 | .27 | .06 | .13 | .23 | .00 |
| Occipital Pole | .27 | .27 | .19 | .09 | .15 | .22 | .24 | .16 | .13 | .28 | .26 | .12 |
| Temporal Occipital Fusiform Cortex | .00 | .24 | .03 | .20 | .00 | .26 | .07 | .04 | .00 | .00 | .00 | .00 |
| Occipital Fusiform Gyrus | .23 | .39 | .00 | .09 | .18 | .18 | .16 | .04 | .00 | .00 | .09 | .00 |
| Cuneal Cortex | .31 | .23 | .00 | .19 | .16 | .41 | .00 | .00 | .00 | .35 | .00 | 1 |
| Lingual Gyrus | .24 | .35 | .11 | .06 | .31 | .21 | .21 | .05 | .01 | .20 | .11 | .16 |
| Intracalcarine Cortex | .10 | .14 | .14 | .00 | .01 | .24 | .30 | .00 | .10 | .68 | .16 | .20 |
| Supracalcarine Cortex | .23 | .23 | .18 | .00 | .04 | .33 | .13 | .00 | .02 | .35 | .00 | .25 |
| Temporal Fusiform Cortex anterior | .20 | .16 | .28 | .00 | .19 | .04 | .65 | .11 | .34 | .49 | .20 | .00 |
| Temporal Pole | .21 | .12 | .11 | .13 | .56 | .23 | .21 | .32 | .08 | .15 | .20 | .09 |
| Middle Temporal Gyrus anterior | .19 | .34 | .06 | .16 | .17 | .22 | .13 | .36 | .32 | .27 | .16 | .19 |
| Inferior Temporal Gyrus anterior | .37 | .26 | .20 | .00 | .28 | .25 | .15 | .30 | .10 | .35 | .33 | .07 |
| Parahippocampal Gyrus posterior | .00 | .33 | .04 | .25 | .23 | .20 | .37 | .00 | .00 | .10 | .00 | .13 |
| Temporal Fusiform Cortex posterior | .00 | .33 | .16 | .48 | .10 | .13 | .14 | .03 | .00 | .13 | .00 | .03 |
| Parahippocampal Gyrus anterior | .41 | .23 | .47 | .00 | .05 | .11 | .22 | .03 | .00 | .34 | .63 | 1 |
| Hippocampus | .09 | .27 | .11 | .00 | .19 | .14 | .41 | .44 | .00 | .03 | .00 | .33 |
| Amygdala | .10 | .39 | .03 | .16 | .26 | .14 | .27 | .15 | .00 | .15 | .23 | .17 |
| Putamen | .32 | .60 | .34 | .00 | .11 | .21 | .43 | .00 | .22 | .19 | .20 | .00 |
| Accumbens | .12 | .23 | .19 | .13 | .10 | .30 | .38 | .00 | .12 | .13 | .15 | .00 |
| Thalamus | .00 | .34 | .08 | .00 | .13 | .14 | .00 | .17 | .00 | .04 | .00 | .12 |
| Caudate | .06 | .31 | .00 | .01 | .11 | .10 | .00 | .11 | .19 | .22 | .40 | .00 |
| Central Opercular Cortex | .10 | .15 | .21 | .20 | .09 | .12 | .28 | .02 | .07 | .28 | .25 | .12 |
| Parietal Operculum Cortex | .02 | .19 | .20 | .00 | .19 | .34 | .29 | .04 | .27 | .36 | .08 | .47 |
| Heschl.s Gyrus | .11 | .23 | .10 | .13 | .16 | .05 | .00 | .18 | .54 | .40 | .43 | .38 |
| Planum Temporale | .14 | .22 | .19 | .23 | .19 | .19 | .10 | .11 | .26 | .35 | .34 | .28 |
| Planum Polare | .00 | .17 | .13 | .00 | .18 | .09 | .09 | .03 | .30 | .55 | .42 | .16 |
| Insular Cortex | .15 | .16 | .22 | .12 | .27 | .17 | .12 | .09 | .08 | .10 | .00 | .00 |
| Frontal Operculum Cortex | .21 | .25 | .25 | .25 | .22 | .10 | .00 | .11 | .18 | .32 | .33 | .00 |
| Paracingulate Gyrus | .15 | .18 | .18 | .05 | .17 | .21 | .34 | .12 | .17 | .25 | .20 | .14 |
| Cingulate Gyrus anterior | .19 | .10 | .35 | .00 | .08 | .21 | .25 | .12 | 1 | .24 | .00 | .10 |
| Subcallosal Cortex | .08 | .24 | .43 | .58 | .10 | .14 | .11 | .05 | .08 | .13 | .00 | .01 |
| Frontal Medial Cortex | .09 | .28 | .67 | .33 | .04 | .24 | .08 | .16 | .08 | .51 | .35 | .00 |
| Frontal Orbital Cortex | .11 | .23 | .24 | .17 | .18 | .28 | .09 | .41 | .00 | .00 | .00 | .00 |
| Mean | .20 | .25 | .20 | .15 | .16 | .20 | .23 | .14 | .13 | .24 | .14 | .13 |
| SD | .13 | .10 | .14 | .14 | .11 | .10 | .16 | .14 | .18 | .17 | .15 | .21 |

**Figure S5.** Comparison of weighted clustering coefficient stratified by brain region, diagnostic group and modality for the partial correlation matrices of the left hemisphere. Averaged over ten repetitions. CN: cognitively healthy elderly controls, EMCI/LMCI: early and late amnestic mild cognitive impairment, AD: Alzheimer's dementia, amy: amyloid-$\beta$, metab: glucose metabolism, vol: gray matter volume.

Characteristic path length by region

| | amy CN | amy EMCI | amy LMCI | amy AD | metab CN | metab EMCI | metab LMCI | metab AD | vol CN | vol EMCI | vol LMCI | vol AD |
|---|---|---|---|---|---|---|---|---|---|---|---|---|
| Inferior Frontal Gyrus pars opercularis | 12.24 | 12.64 | 10.92 | 11.78 | 13.22 | 12.84 | 10.34 | 11.72 | 13.36 | 14.22 | 14.75 | 12.87 |
| Inferior Frontal Gyrus pars triangularis | 12.33 | 12.69 | 10.86 | 12.16 | 12.72 | 12.22 | 10.97 | 12.26 | 13.01 | 12.53 | 12.86 | 13.25 |
| Frontal Pole | 11.71 | 13.01 | 10.05 | 12.32 | 11.15 | 11.20 | 11.77 | 12.22 | 12.75 | 13.87 | 13.19 | 11.74 |
| Middle Frontal Gyrus | 12.54 | 13.42 | 11.01 | 12.83 | 11.43 | 10.85 | 11.69 | 11.84 | 14.67 | 14.90 | 13.36 | 11.08 |
| Superior Temporal Gyrus anterior | 11.73 | 12.10 | 10.88 | 13.48 | 10.61 | 10.84 | 10.43 | 10.42 | 13.26 | 13.26 | 11.92 | 12.82 |
| Superior Temporal Gyrus posterior | 10.79 | 11.28 | 10.32 | 11.94 | 11.68 | 10.08 | 10.31 | 11.10 | 12.27 | 12.42 | 12.10 | 12.07 |
| Inferior Temporal Gyrus temporooccipital | 13.44 | 11.79 | 10.97 | 11.91 | 10.52 | 11.01 | 10.15 | 12.16 | 13.40 | 14.04 | 12.20 | 12.38 |
| Inferior Temporal Gyrus posterior | 12.78 | 12.19 | 10.72 | 11.66 | 11.56 | 11.33 | 10.18 | 11.78 | 12.94 | 13.44 | 11.69 | 12.11 |
| Middle Temporal Gyrus posterior | 11.35 | 11.53 | 9.96 | 11.86 | 12.31 | 10.99 | 10.56 | 11.11 | 12.74 | 12.10 | 11.99 | 11.44 |
| Middle Temporal Gyrus temporooccipital | 12.14 | 11.62 | 11.73 | 11.72 | 12.18 | 11.76 | 11.61 | 11.84 | 13.22 | 13.34 | 13.10 | 11.13 |
| Supramarginal Gyrus anterior | 12.27 | 12.00 | 12.61 | 12.21 | 10.71 | 10.14 | 9.92 | 11.87 | 13.11 | 13.60 | 12.92 | 12.70 |
| Lateral Occipital Cortex superior | 12.16 | 11.36 | 10.90 | 11.85 | 11.34 | 10.41 | 11.27 | 12.30 | 12.82 | 12.21 | 13.32 | 10.96 |
| Supramarginal Gyrus posterior | 11.73 | 11.65 | 11.96 | 12.32 | 10.97 | 11.05 | 10.03 | 11.71 | 13.19 | 13.12 | 12.31 | 12.86 |
| Angular Gyrus | 12.05 | 11.68 | 10.96 | 12.24 | 11.93 | 12.16 | 10.26 | 11.88 | 13.73 | 13.39 | 12.93 | 13.03 |
| Cingulate Gyrus posterior | 13.13 | 11.93 | 12.34 | 12.05 | 12.66 | 11.33 | 10.82 | 12.66 | 12.99 | 12.78 | 14.16 | 13.32 |
| Precuneus Cortex | 12.68 | 11.17 | 12.33 | 11.38 | 12.07 | 11.74 | 10.70 | 12.36 | 12.66 | 13.67 | 14.86 | 12.38 |
| Superior Frontal Gyrus | 14.09 | 13.05 | 12.21 | 12.57 | 11.68 | 11.66 | 11.46 | 12.67 | 12.94 | 13.56 | 11.22 | 12.47 |
| Juxtapositional Lobule Cortex | 14.42 | 12.35 | 11.33 | 11.44 | 11.88 | 12.01 | 11.81 | 12.31 | 13.35 | 14.76 | 13.51 | 11.65 |
| Superior Parietal Lobule | 12.30 | 12.89 | 13.41 | 11.58 | 12.22 | 11.79 | 11.02 | 13.11 | 13.56 | 13.69 | 15.65 | 11.87 |
| Precentral Gyrus | 13.03 | 12.17 | 10.51 | 10.45 | 11.75 | 10.79 | 11.39 | 11.65 | 12.09 | 12.70 | 11.86 | 10.74 |
| Postcentral Gyrus | 12.21 | 12.11 | 11.05 | 11.02 | 11.01 | 10.03 | 10.61 | 12.02 | 12.01 | 12.02 | 12.89 | 10.75 |
| Lateral Occipital Cortex inferior | 11.71 | 11.30 | 11.07 | 11.63 | 11.17 | 10.89 | 11.77 | 11.71 | 12.10 | 12.54 | 12.23 | 10.74 |
| Occipital Pole | 11.36 | 11.04 | 12.74 | 11.13 | 15.80 | 11.55 | 13.21 | 11.80 | 12.38 | 12.92 | 11.81 | 12.10 |
| Temporal Occipital Fusiform Cortex | 11.89 | 12.93 | 12.34 | 12.83 | 12.52 | 10.68 | 11.32 | 11.93 | 12.42 | 14.26 | 12.75 | 11.45 |
| Occipital Fusiform Gyrus | 12.13 | 13.02 | 12.22 | 12.80 | 12.22 | 10.14 | 11.40 | 12.23 | 13.12 | 14.47 | 13.11 | 11.02 |
| Cuneal Cortex | 13.72 | 12.17 | 16.60 | 13.46 | 12.94 | 13.28 | 14.00 | 14.17 | 14.42 | 15.10 | 15.74 | 13.63 |
| Lingual Gyrus | 12.72 | 13.60 | 12.64 | 13.79 | 12.15 | 9.68 | 9.51 | 12.34 | 14.89 | 15.47 | 13.36 | 11.32 |
| Intracalcarine Cortex | 12.75 | 13.95 | 14.35 | 14.57 | 12.73 | 11.95 | 12.19 | 14.23 | 15.52 | 15.59 | 12.69 | 13.29 |
| Supracalcarine Cortex | 13.72 | 13.49 | 15.37 | 14.25 | 12.71 | 12.76 | 12.75 | 14.46 | 14.61 | 14.74 | 14.36 | 13.87 |
| Temporal Fusiform Cortex anterior | 13.28 | 13.37 | 12.25 | 13.84 | 13.61 | 12.99 | 11.26 | 13.23 | 13.25 | 13.90 | 13.00 | 12.47 |
| Temporal Pole | 12.38 | 12.12 | 11.90 | 12.04 | 13.38 | 12.04 | 11.01 | 12.33 | 11.83 | 12.81 | 12.96 | 11.81 |
| Middle Temporal Gyrus anterior | 11.60 | 11.89 | 11.02 | 12.52 | 12.52 | 11.33 | 10.93 | 11.69 | 12.87 | 12.61 | 13.29 | 12.06 |
| Inferior Temporal Gyrus anterior | 13.32 | 13.89 | 13.32 | 13.05 | 12.45 | 12.08 | 12.17 | 12.27 | 13.02 | 13.90 | 13.08 | 12.08 |
| Parahippocampal Gyrus posterior | 14.26 | 12.56 | 10.91 | 13.04 | 11.78 | 10.21 | 11.06 | 13.63 | 14.89 | 15.55 | 13.77 | 11.71 |
| Temporal Fusiform Cortex posterior | 12.79 | 12.62 | 10.53 | 12.33 | 10.90 | 10.33 | 10.58 | 11.68 | 11.72 | 12.47 | 11.27 | 10.57 |
| Parahippocampal Gyrus anterior | 14.08 | 14.29 | 12.39 | 16.26 | 12.43 | 12.64 | 11.82 | 12.91 | 13.38 | 15.52 | 15.42 | 13.70 |
| Hippocampus | 14.20 | 12.81 | 11.95 | 15.23 | 12.01 | 10.46 | 10.19 | 12.14 | 14.32 | 15.28 | 16.35 | 13.00 |
| Amygdala | 14.49 | 12.78 | 14.22 | 15.28 | 11.42 | 10.72 | 10.13 | 11.76 | 13.08 | 14.89 | 16.24 | 12.70 |
| Putamen | 15.18 | 13.87 | 13.58 | 13.24 | 13.67 | 12.47 | 10.04 | 13.54 | 13.75 | 14.73 | 12.67 | 14.77 |
| Accumbens | 13.40 | 11.19 | 11.87 | 12.11 | 12.63 | 13.15 | 11.87 | 12.41 | 13.88 | 14.85 | 13.21 | 13.53 |
| Thalamus | 15.45 | 15.64 | 13.16 | 14.54 | 10.60 | 11.25 | 12.09 | 10.91 | 14.81 | 14.92 | 15.70 | 13.14 |
| Caudate | 14.51 | 11.98 | 12.80 | 13.40 | 11.92 | 10.79 | 13.52 | 12.04 | 13.78 | 14.20 | 14.90 | 13.35 |
| Central Opercular Cortex | 12.85 | 11.23 | 12.13 | 11.61 | 11.21 | 11.23 | 10.30 | 11.90 | 11.99 | 12.54 | 12.83 | 11.50 |
| Parietal Operculum Cortex | 12.70 | 12.00 | 11.19 | 12.66 | 11.57 | 11.07 | 10.19 | 13.61 | 12.36 | 13.12 | 13.66 | 13.70 |
| Heschl.s Gyrus | 12.46 | 11.89 | 11.21 | 12.16 | 10.66 | 10.92 | 11.76 | 13.61 | 13.25 | 12.14 | 12.61 | 12.65 |
| Planum Temporale | 11.94 | 11.28 | 11.01 | 12.41 | 11.63 | 10.96 | 11.61 | 13.08 | 12.71 | 12.70 | 12.79 | 12.92 |
| Planum Polare | 13.18 | 12.46 | 11.04 | 13.42 | 10.58 | 10.82 | 10.09 | 11.36 | 12.64 | 12.54 | 12.81 | 11.64 |
| Insular Cortex | 12.11 | 11.93 | 11.89 | 11.36 | 11.90 | 11.12 | 10.54 | 11.16 | 11.00 | 12.04 | 12.00 | 12.39 |
| Frontal Operculum Cortex | 12.27 | 11.32 | 11.38 | 10.43 | 12.96 | 11.81 | 12.81 | 11.36 | 11.81 | 12.23 | 12.73 | 13.40 |
| Paracingulate Gyrus | 11.72 | 12.26 | 10.79 | 11.27 | 10.48 | 10.90 | 12.21 | 12.05 | 12.57 | 14.06 | 14.96 | 12.04 |
| Cingulate Gyrus anterior | 12.66 | 12.19 | 11.55 | 11.55 | 10.33 | 11.14 | 11.92 | 11.99 | 15.65 | 14.07 | 17.87 | 15.94 |
| Subcallosal Cortex | 12.84 | 12.07 | 13.15 | 12.74 | 11.89 | 13.62 | 12.53 | 11.99 | 13.79 | 14.46 | 14.30 | 13.31 |
| Frontal Medial Cortex | 12.71 | 13.06 | 14.33 | 12.73 | 11.03 | 12.26 | 12.80 | 12.58 | 12.73 | 14.97 | 14.24 | 13.12 |
| Frontal Orbital Cortex | 12.38 | 11.59 | 11.04 | 11.37 | 13.94 | 12.15 | 11.50 | 12.48 | 12.56 | 12.97 | 13.21 | 13.05 |
| Mean | 12.78 | 12.38 | 11.94 | 12.52 | 11.95 | 11.40 | 11.27 | 12.25 | 13.17 | 13.67 | 13.42 | 12.44 |
| SD | 1.03 | .93 | 1.35 | 1.23 | 1.05 | .91 | 1.02 | .89 | .98 | 1.09 | 1.40 | 1.10 |

**Figure S6.** Comparison of characteristic path length stratified by brain region, diagnostic group and modality for the partial correlation matrices of the left hemisphere. Averaged over ten repetitions. CN: cognitively healthy elderly controls, EMCI/LMCI: early and late amnestic mild cognitive impairment, AD: Alzheimer's dementia, amy: amyloid-$\beta$, metab: glucose metabolism, vol: gray matter volume.

Small−world coefficient by region

| | amy CN | amy EMCI | amy LMCI | amy AD | metab CN | metab EMCI | metab LMCI | metab AD | vol CN | vol EMCI | vol LMCI | vol AD |
|---|---|---|---|---|---|---|---|---|---|---|---|---|
| Inferior Frontal Gyrus pars opercularis | 13.01 | 13.90 | 10.92 | 10.34 | 10.58 | 1.85 | 3.79 | 22.33 | 14.32 | 22.39 | .00 | .00 |
| Inferior Frontal Gyrus pars triangularis | 16.63 | 6.07 | 27.30 | .00 | .00 | 7.62 | 16.72 | .00 | 8.32 | 17.85 | 13.05 | .00 |
| Frontal Pole | 17.98 | 11.95 | 24.99 | 8.95 | 14.55 | 22.66 | 29.67 | 1.18 | .00 | 5.81 | 17.10 | 7.03 |
| Middle Frontal Gyrus | 23.03 | 20.91 | 21.42 | 8.67 | 11.90 | 16.99 | 20.75 | .00 | 25.55 | 44.45 | 2.47 | 9.38 |
| Superior Temporal Gyrus anterior | 10.33 | 15.45 | 6.00 | 23.29 | 22.77 | 13.30 | 7.47 | 14.05 | .00 | 5.31 | .00 | .00 |
| Superior Temporal Gyrus posterior | 15.87 | 17.50 | 14.66 | 11.30 | 11.56 | 18.39 | 20.65 | 12.61 | 2.47 | 27.21 | 27.00 | 8.68 |
| Inferior Temporal Gyrus temporooccipital | 8.90 | 14.97 | 15.30 | 18.67 | 8.38 | 12.51 | 21.35 | 4.87 | .00 | 41.81 | 18.63 | .00 |
| Inferior Temporal Gyrus posterior | .00 | 13.80 | 26.82 | 21.82 | 8.25 | 16.66 | 13.92 | 7.22 | .00 | 16.92 | 13.10 | .00 |
| Middle Temporal Gyrus posterior | 15.40 | 9.74 | 21.70 | 15.71 | .00 | 11.08 | 12.75 | 15.17 | 15.54 | 17.31 | .56 | 1.67 |
| Middle Temporal Gyrus temporooccipital | 20.00 | 24.41 | 12.10 | .00 | 19.03 | 50.11 | 40.54 | 46.49 | .00 | .00 | .00 | 2.94 |
| Supramarginal Gyrus anterior | 20.61 | 26.06 | 23.93 | 16.36 | 29.13 | 18.10 | 16.26 | 15.43 | 9.43 | 33.21 | 11.66 | .00 |
| Lateral Occipital Cortex superior | 33.82 | 37.66 | 11.74 | 16.51 | 13.38 | 17.23 | 22.36 | 12.92 | 22.67 | 6.83 | .00 | 24.85 |
| Supramarginal Gyrus posterior | 33.38 | 15.30 | 1.63 | 32.67 | 23.89 | 21.46 | 46.56 | 7.77 | .00 | 17.62 | 10.55 | .00 |
| Angular Gyrus | 29.24 | 28.46 | 12.06 | 17.13 | 20.12 | 9.70 | 44.97 | 11.37 | 11.19 | .00 | .00 | 4.48 |
| Cingulate Gyrus posterior | 23.75 | 27.22 | 40.43 | 27.16 | 10.01 | 13.85 | 40.71 | 26.04 | .00 | 10.93 | 1.64 | .00 |
| Precuneus Cortex | 26.21 | 25.59 | 13.12 | 17.15 | 6.94 | 28.27 | 48.49 | 19.96 | .00 | 15.26 | .00 | 7.10 |
| Superior Frontal Gyrus | 25.96 | 11.82 | 27.87 | .00 | 16.10 | 26.48 | 33.74 | .00 | 12.07 | 15.05 | 8.98 | 6.06 |
| Juxtapositional Lobule Cortex | 16.36 | 10.46 | 23.74 | 16.81 | 15.38 | 18.32 | 21.97 | .44 | 22.61 | 26.74 | 29.20 | 26.70 |
| Superior Parietal Lobule | 40.05 | 15.02 | 29.51 | 14.26 | 35.69 | 24.09 | 48.52 | 16.84 | .00 | .00 | .00 | 20.81 |
| Precentral Gyrus | 28.29 | 22.98 | 13.23 | 18.53 | 8.81 | 19.81 | 26.35 | 22.44 | 9.05 | 13.90 | .00 | 16.56 |
| Postcentral Gyrus | 24.82 | 20.99 | 19.38 | 33.24 | 11.49 | 26.93 | 15.86 | 29.16 | 16.28 | 10.29 | .00 | 2.95 |
| Lateral Occipital Cortex inferior | 23.85 | 35.85 | 21.73 | 9.32 | 15.40 | 32.61 | 27.65 | 23.09 | 5.26 | 10.48 | 18.45 | .00 |
| Occipital Pole | 23.40 | 24.43 | 14.90 | 8.21 | 9.22 | 19.41 | 17.96 | 13.83 | 10.74 | 21.84 | 22.26 | 10.16 |
| Temporal Occipital Fusiform Cortex | .00 | 18.73 | 2.57 | 15.56 | .00 | 24.04 | 6.33 | 3.12 | .00 | .00 | .00 | .00 |
| Occipital Fusiform Gyrus | 19.06 | 30.01 | .00 | 6.84 | 14.67 | 17.39 | 13.72 | 3.35 | .00 | .00 | 7.23 | .00 |
| Cuneal Cortex | 22.64 | 19.24 | .00 | 13.90 | 12.17 | 31.01 | .00 | .00 | .00 | 22.88 | .00 | 73.39 |
| Lingual Gyrus | 18.72 | 25.40 | 8.86 | 4.62 | 25.51 | 21.52 | 21.73 | 4.32 | .92 | 12.94 | 8.61 | 14.39 |
| Intracalcarine Cortex | 7.80 | 9.94 | 9.79 | .00 | .62 | 20.48 | 24.95 | .00 | 6.07 | 43.61 | 12.27 | 15.86 |
| Supracalcarine Cortex | 17.03 | 16.83 | 11.61 | .00 | 3.42 | 26.12 | 10.26 | .00 | 1.08 | 23.42 | .00 | 18.16 |
| Temporal Fusiform Cortex anterior | 14.79 | 12.34 | 22.90 | .00 | 13.93 | 2.86 | 57.93 | 8.25 | 25.49 | 35.01 | 15.52 | .00 |
| Temporal Pole | 16.90 | 9.52 | 9.32 | 10.48 | 41.77 | 19.17 | 19.40 | 25.82 | 6.86 | 11.50 | 15.29 | 7.52 |
| Middle Temporal Gyrus anterior | 16.16 | 28.74 | 5.86 | 12.56 | 13.38 | 19.20 | 11.60 | 30.47 | 24.60 | 21.25 | 12.26 | 15.86 |
| Inferior Temporal Gyrus anterior | 27.84 | 19.01 | 15.32 | .00 | 22.70 | 20.79 | 12.10 | 24.69 | 7.59 | 25.52 | 25.49 | 6.15 |
| Parahippocampal Gyrus posterior | .00 | 26.39 | 3.98 | 19.61 | 19.62 | 19.38 | 33.20 | .00 | .00 | 6.35 | .00 | 11.49 |
| Temporal Fusiform Cortex posterior | .00 | 25.93 | 15.49 | 39.24 | 8.97 | 12.62 | 12.98 | 2.30 | .00 | 10.06 | .00 | 2.80 |
| Parahippocampal Gyrus anterior | 29.46 | 15.71 | 38.01 | .00 | 3.87 | 9.08 | 18.55 | 2.15 | .00 | 22.05 | 40.86 | 72.98 |
| Hippocampus | 6.31 | 20.78 | 9.59 | .00 | 15.75 | 13.83 | 40.37 | 36.53 | .00 | 1.72 | .00 | 25.31 |
| Amygdala | 7.12 | 30.87 | 2.22 | 10.48 | 22.83 | 12.80 | 26.71 | 13.16 | .00 | 10.39 | 14.34 | 13.07 |
| Putamen | 21.14 | 43.58 | 24.85 | .00 | 7.65 | 16.57 | 42.57 | .00 | 15.71 | 12.85 | 15.58 | .00 |
| Accumbens | 8.72 | 20.61 | 15.93 | 10.87 | 7.99 | 22.91 | 31.95 | .00 | 8.76 | 8.69 | 11.07 | .00 |
| Thalamus | .00 | 21.58 | 6.08 | .00 | 11.89 | 12.88 | .00 | 15.92 | .00 | 2.78 | .00 | 9.68 |
| Caudate | 4.27 | 26.16 | .00 | .72 | 9.45 | 9.22 | .00 | 9.13 | 14.04 | 15.41 | 26.82 | .00 |
| Central Opercular Cortex | 7.86 | 13.55 | 17.70 | 17.04 | 7.75 | 10.95 | 26.75 | 1.86 | 5.59 | 22.02 | 19.19 | 10.71 |
| Parietal Operculum Cortex | 1.91 | 15.63 | 17.58 | .00 | 16.11 | 30.69 | 28.09 | 3.25 | 22.10 | 27.11 | 5.94 | 33.75 |
| Heschl.s Gyrus | 8.74 | 19.49 | 8.71 | 10.32 | 14.78 | 4.75 | .00 | 13.35 | 40.66 | 33.36 | 34.30 | 30.27 |
| Planum Temporale | 11.86 | 19.80 | 17.14 | 18.22 | 16.57 | 17.10 | 8.76 | 8.56 | 20.60 | 27.57 | 26.58 | 21.44 |
| Planum Polare | .00 | 13.42 | 11.60 | .00 | 17.46 | 8.25 | 8.82 | 3.03 | 23.64 | 44.12 | 32.25 | 14.13 |
| Insular Cortex | 12.64 | 13.23 | 18.69 | 10.63 | 22.59 | 15.44 | 11.83 | 8.20 | 6.98 | 8.51 | .00 | .00 |
| Frontal Operculum Cortex | 17.39 | 22.36 | 22.01 | 24.18 | 17.00 | 8.88 | .00 | 9.35 | 15.28 | 26.27 | 25.79 | .00 |
| Paracingulate Gyrus | 13.14 | 14.51 | 16.22 | 4.38 | 16.59 | 18.98 | 27.81 | 9.74 | 13.30 | 18.09 | 13.09 | 11.22 |
| Cingulate Gyrus anterior | 15.14 | 8.24 | 29.90 | .00 | 7.55 | 18.82 | 21.13 | 9.74 | 63.92 | 17.42 | .00 | 6.04 |
| Subcallosal Cortex | 6.27 | 19.96 | 32.96 | 45.66 | 8.22 | 9.95 | 8.85 | 3.75 | 5.58 | 8.66 | .00 | .48 |
| Frontal Medial Cortex | 7.07 | 21.11 | 47.05 | 25.73 | 3.79 | 19.58 | 5.88 | 13.04 | 6.35 | 34.22 | 24.70 | .00 |
| Frontal Orbital Cortex | 8.93 | 19.82 | 21.47 | 15.28 | 13.24 | 23.41 | 7.71 | 33.16 | .00 | .00 | .00 | .00 |
| Mean | 15.55 | 19.87 | 16.63 | 12.27 | 13.71 | 17.89 | 21.09 | 11.47 | 9.64 | 17.32 | 10.77 | 10.45 |
| SD | 10.27 | 8.15 | 10.83 | 11.28 | 8.75 | 8.28 | 14.75 | 11.72 | 12.46 | 12.35 | 11.36 | 15.71 |

**Figure S7.** Comparison of small-world coefficient stratified by brain region, diagnostic group and modality for the partial correlation matrices of the left hemisphere. For better readability, individual values were upscaled by a factor of 1,000. Averaged over ten repetitions.
CN: cognitively healthy elderly controls, EMCI/LMCI: early and late amnestic mild cognitive impairment, AD: Alzheimer's dementia, amy: amyloid-$\beta$, metab: glucose metabolism, vol: gray matter volume.

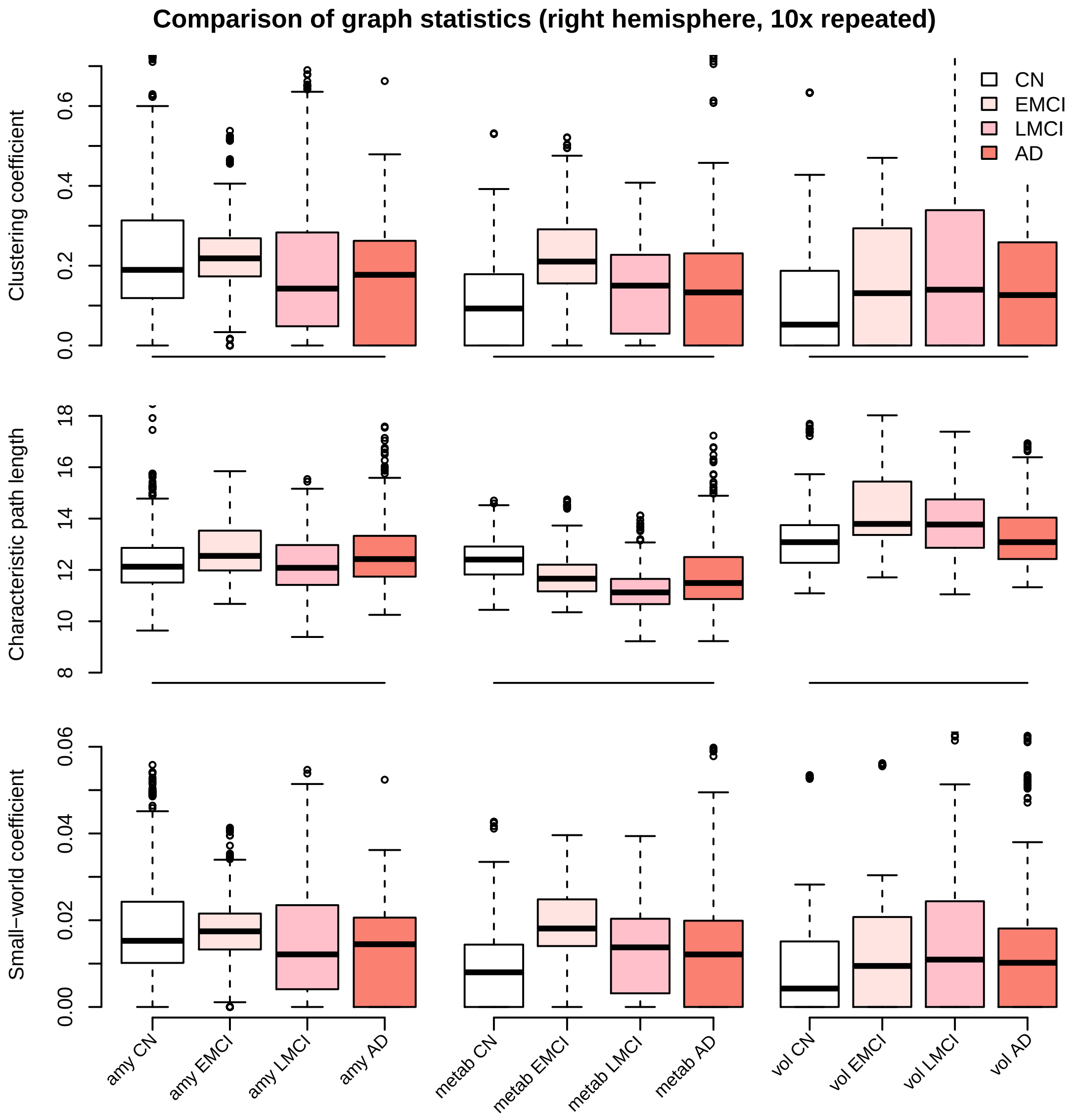


**Figure S8.** Comparison of graph statistics for the partial correlation matrices of the **right** hemisphere stratified by diagnostic group and image modality. Estimates based on Gaussian graphical models using multimodal neuroimaging data. The distribution of the weighted clustering coefficient, characteristic weighted path length, and small-world coefficient for individual brain regions is shown. Boxes display median, first and third quartile of the distributions, and whiskers indicate $\pm 1.5\times$interquartile range.
CN: cognitively healthy elderly controls, EMCI/LMCI: early and late amnestic mild cognitive impairment, AD: Alzheimer's dementia, amy: amyloid-$\beta$, metab: glucose metabolism, vol: gray matter volume.

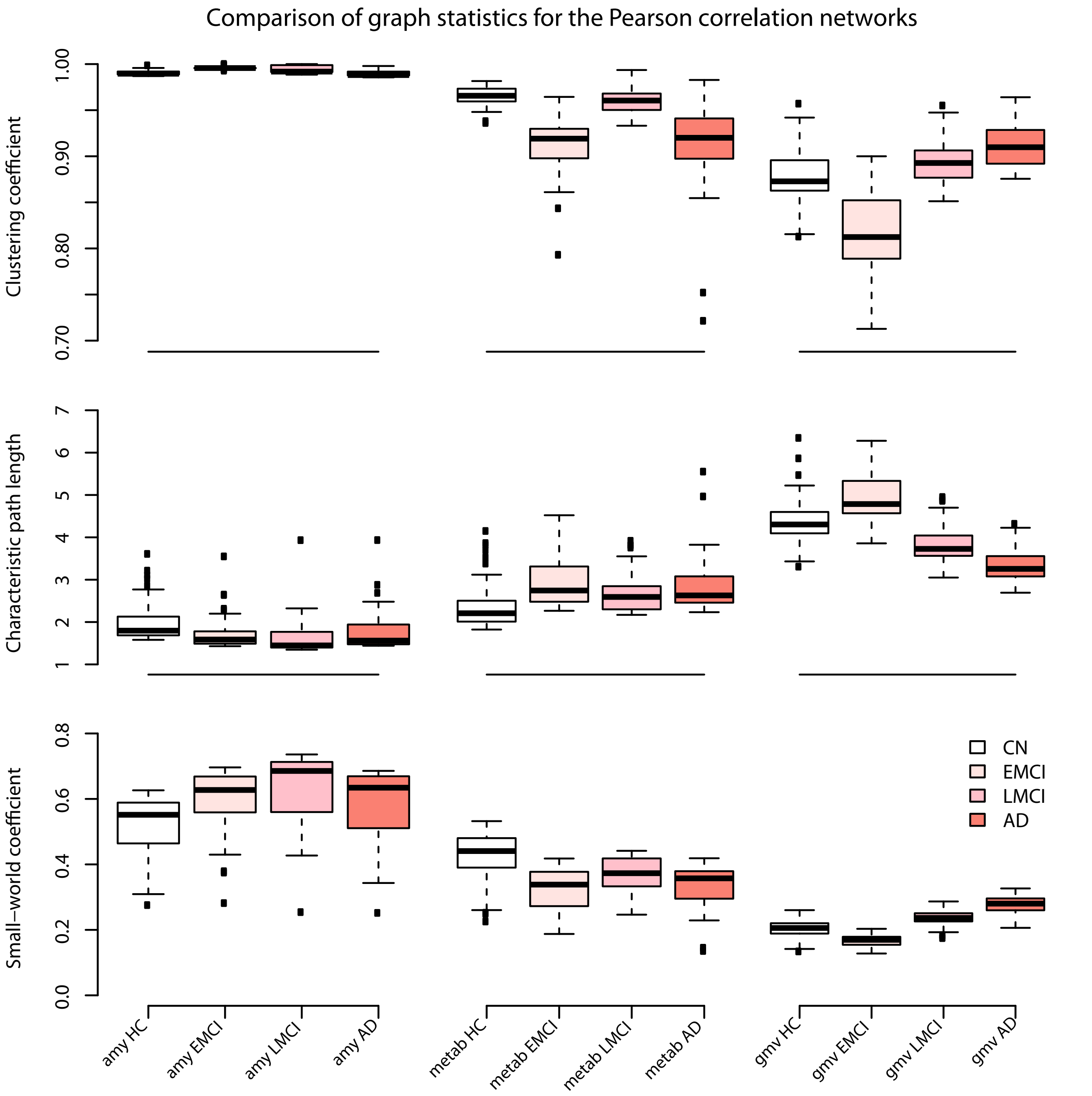


**Figure S9.** Comparison of graph statistics for the Pearson correlation matrices of the left hemisphere stratified by diagnostic group and image modality. The distribution of the weighted clustering coefficient, characteristic weighted path length, and small-world coefficient for individual brain regions is shown. Boxes display median, first and third quartile of the distributions, and whiskers indicate $\pm 1.5\times$interquartile range. Prior to calculating the graph measures, the correlation matrices were thresholded such that correlations with $p > 0.05$, i.e. approximately $r < 0.12$, were set to zero.
CN: cognitively healthy elderly controls, EMCI/LMCI: early and late amnestic mild cognitive impairment, AD: Alzheimer's dementia, amy: amyloid-$\beta$, metab: glucose metabolism, vol: gray matter volume.

**Table S1.** P-values for the comparison of graph statistics based on Pearson correlation (Figure S9).

| | | Amyloid-$\beta$ | | | Metabolism | | | Volume | | |
|---|---|---|---|---|---|---|---|---|---|---|
| | | EMCI | LMCI | AD | EMCI | LMCI | AD | EMCI | LMCI | AD |
| Clustering coefficient | CN | < 0.001 | < 0.001 | 0.575 | < 0.001 | 0.759 | < 0.001 | < 0.001 | 0.103 | < 0.001 |
| | EMCI | | < 0.001 | < 0.001 | | < 0.001 | 0.973 | | < 0.001 | < 0.001 |
| | LMCI | | | < 0.001 | | | < 0.001 | | | 0.020 |
| Path length | CN | 0.013 | < 0.001 | 0.026 | < 0.001 | 0.051 | < 0.001 | < 0.001 | < 0.001 | < 0.001 |
| | EMCI | | 0.437 | 0.996 | | 0.113 | 0.857 | | < 0.001 | < 0.001 |
| | LMCI | | | 0.315 | | | 0.464 | | | < 0.001 |
| Small-world coefficient | CN | < 0.001 | < 0.001 | 0.001 | < 0.001 | < 0.001 | < 0.001 | < 0.001 | < 0.001 | < 0.001 |
| | EMCI | | 0.085 | 0.991 | | 0.004 | 0.797 | | < 0.001 | < 0.001 |
| | LMCI | | | 0.040 | | | 0.055 | | | < 0.001 |

Adjusted P-values from Tukey's honest significant difference tests, controlling for family-wise error rate within each comparison block. CN: cognitively normal controls, EMCI/LMCI: early/late amnestic mild cognitive impairment, AD: Alzheimer's dementia.

**Table S2.** Analysis of variance (ANOVA) results for the graph statistics for the partial correlation networks in Figure 9.

| | | F-statistic | P-value | Effect size $\eta^2$ |
|---|---|---|---|---|
| Clustering coefficient | amy | 5.6 | 0.001 | 0.07 |
| | metab | 6.2 | < 0.001 | 0.08 |
| | vol | 4.6 | 0.004 | 0.06 |
| Characteristic path length | amy | 5.1 | 0.002 | 0.07 |
| | metab | 12.8 | < 0.001 | 0.15 |
| | vol | 11.8 | < 0.001 | 0.14 |
| Small-world coefficient | amy | 5.6 | 0.001 | 0.07 |
| | metab | 8.6 | < 0.001 | 0.11 |
| | vol | 4.1 | 0.007 | 0.06 |

df=215 for all models, amy: amyloid-$\beta$, metab: glucose metabolism, vol: gray matter volume.
